# A unifying view on extended phase graphs and Bloch simulations for quantitative MRI

**DOI:** 10.1038/s41598-021-00233-6

**Published:** 2021-10-28

**Authors:** Christian Guenthner, Thomas Amthor, Mariya Doneva, Sebastian Kozerke

**Affiliations:** 1 Institute for Biomedical Engineering, University and ETH Zurich, Zurich, Switzerland; 2 Philips Research, Hamburg, Germany

**Keywords:** Biomedical engineering, Applied physics

## Abstract

Quantitative MRI methods and learning-based algorithms require exact forward simulations. One critical factor to correctly describe magnetization dynamics is the effect of slice-selective RF pulses. While contemporary simulation techniques correctly capture their influence, they only provide final magnetization distributions, require to be run for each parameter set separately, and make it hard to derive general theoretical conclusions and to generate a fundamental understanding of echo formation in the presence of slice-profile effects. This work aims to provide a mathematically exact framework, which is equally intuitive as extended phase graphs (EPGs), but also considers slice-profiles through their natural spatial representation. We show, through an analytical, hybrid Bloch-EPG formalism, that the spatially-resolved EPG approach allows to exactly predict the signal dependency on off-resonance, spoiling moment, microscopic dephasing, and echo time. We also demonstrate that our formalism allows to use the same phase graph to simulate both gradient-spoiled and balanced SSFP-based MR sequences. We present a derivation of the formalism and identify the connection to existing methods, i.e. slice-selective Bloch, slice-selective EPG, and the partitioned EPG. As a use case, the proposed hybrid Bloch-EPG framework is applied to MR Fingerprinting.

## Introduction

In recent years, interest in MR sequence simulations has risen due to the advent of MR Fingerprinting (MRF) and the need for synthetic data to train neural networks^1–5^. While all simulation approaches solve the Bloch equations^6^ in one or another form, two main classes of techniques can be distinguished: (1) direct solution of the Bloch equations in the spatial domain (here simply called “Bloch simulations”), which can be performed through consecutive application of matrix operators (rotation operator algorithm^7^) or via general numerical differential equation solvers; or (2) indirect solution in the Fourier domain using extended phase graphs (EPG)^8^. While the former is especially useful for balanced SSFP simulations and general MR simulators (e.g. JEMRIS^9^ or the EMC Platform for MESE^10^), the latter is used as the work horse technique whenever spoiling gradients are employed. EPGs are not only numerically stable and computationally efficient, they also provide fundamental insights into MR sequences in terms of dephasing and rephasing configuration pathways, which can interfere and thus modify observed echoes^11^. Extensions to the EPG, such as the spatially-resolved EPG (SR-EPG)^12–14^, EPGs with magnetization transfer and exchange (EPG-X)^15^, EPGs with anisotropic diffusion^16^, three-dimensional EPG^17^, or slice-selective EPGs (ssEPG)^18^ are examples of the continued efforts to improve signal modelling in conjunction with spoiled sequences.

Due to the recent departure from classical, well-understood MR sequences to sequences exploiting the full spectrum of transient magnetization dynamics, fundamental effects resurface in form of confounders. This inspired re-evaluation of e.g. transmit field inhomogeneity ($\Delta {B}_{1}^{+}$), diffusion, slice profiles, intra-voxel dephasing, or magnetic field inhomogenieties ($\Delta {B}_{0}$) in the context of MR Fingerprinting^18–25^.

Especially the problem of slice profiles has recently received interest, as it is straightforward to solve in balanced SSFP-based MRF via Bloch simulations. However, in the context of spoiled sequences, such an approach becomes numerically instable or intractable. Moreover, slice profiles have been identified to re-introduce off-resonance dependencies in gradient-spoiled MRF (FISP-MRF). Effective descriptions employing a variant of the SR-EPG have been employed, which resolve the slice profile by independently simulating a full EPG in each position and then summing along the slice dimension (partitioned EPG, pEPG)^13,14,18^. However, this strategy is only exact in the case of a perfectly homogenous material of infinite or rectangular extent and thus fails to simulate off-resonance artifacts encountered experimentally. This shortcoming has recently been rectified by the introduction of the ssEPG, where the hard pulse approximation is applied directly in the EPG, thus leading to a pure k-space description of the *combined* slice-excitation and spoiling problem^18^. However, this approach necessitates the introduction of surrogate states to describe the effect of the RF pulse. These states are no longer separated by the spoiling moment, but by an arbitrary *discretization moment* arising from the time resolution of the RF pulse simulation. The EPG branches at every discretization point leading to a plethora of configuration pathways, which are no longer easily analyzed in view of echo formation. Thus, the elegant yet simplistic picture of configuration states shifted by spoiling gradients and their interference through RF pulses has been greatly complicated.

In 1994, Sobol and Gauntt^26^ described in detail the magnetization response to gradient-echo sequences with constant repetition time and gradient spoiling moment. They identified interference of substates (later referred to as configuration states) to be accountable for artifacts in gradient-echo sequences. While they worked with isolated, regular prisms (voxels), they noted that slice profiles are rarely rectangular and thus precise, a priori calculation of their effect on the interference of substates was intractable. Despite this early work, considerable misunderstanding of spoiled states in gradient echo sequences has persisted in the community (e.g. the notion of “killer gradients” destroying transverse magnetization) and effects such as off-resonance artifacts in FISP-MRF came as a surprise (in defense of all authors cited and fellow researchers who fell for this: they also came as a surprise to us)^18,22,23^.

In this work, we present a contemporary solution to Sobol’s and Gauntt’s approach for sequences employing arbitrary flip angle trains, which is derived from the rotation operator algorithm that forms the basis of current EPG implementations^8,12,18^. We will work as far as possible with the nomenclature by Weigel^8^ and Malik et al.^12^. Where applicable, crosslinks to the work of Sobol and Gauntt are included^26^.

Our formalism is a hybrid between a conventional spatial Bloch simulation and an extended phase graph: it represents spoiling through configuration states and slice profiles in their natural spatial basis. This allows us to retain the elegance of the EPG formalism which enables the straight-forward identification of echo formation, while keeping the intuition of a spatial basis for the description of slice profiles. Moreover, the formalism is easily implemented in contemporary EPG codes and provides analytical access to multiple key signal dependencies: We show that the signal ${s}^{\left(n\right)}$ for each time-point $n$ in an arbitrary gradient-echo sequence with constant $TR$ and fixed spoiling moment ${k}_{sp}$ is given by a weighted sum over spatially-resolved configurations ${F}_{k}^{+\left(n\right)}(z)$. Here, $k$ is the configuration order and $z$ the spatial position along the slice profile. The signal is given by

1 $${s}^{\left(n\right)}= \sum \limits_{k}{W}_{k}\left(\omega , {R}_{2}^{{\prime}},TE\right)\int {\text{d}}z \, {\text{e}}^{{\text{i}}k{k}_{sp}z}{C\left(z\right){M}_{0}(z) F}_{k}^{+\left(n\right)}(z),$$

where $C(z)$ is the coil sensitivity, ${M}_{0}(z)$ is the equilibrium magnetization and ${W}_{k}(\omega ,{R}_{2}^{{\prime}},TE)$ is an analytical weighting function that captures dependencies on off-resonance $\omega$ and microscopic dephasing ${R}_{2}^{{\prime}}$ for arbitrary echo times $TE$ (see the “Theory” section for the derivation). Through the exponential weighting factor ${\text{e}}^{{\text{i}}k{k}_{sp}z}$, the signal’s dependency on the spoiling moment ${k}_{sp}$ is also obtained analytically. An approximation of the spatially resolved configuration states ${F}_{k}^{+\left(n\right)}\left(z\right)$ can be readily obtained by piecewise discretization of the slice profile and independent EPG simulations (spatially resolved EPG).

We identify balanced steady-state free-precession (bSSFP or True-FISP) sequences as a special case of spoiled (FISP) SSFP by considering ${k}_{sp}=0$. For spoiled SSFP, we recover the partitioned EPG approximation by the limit ${k}_{sp}\to \infty$. Omitting spatial integration, the hybrid Bloch-EPG yields results equal to a direct Bloch simulation. Additional Fourier transformation allows to recover the slice-selective EPG solution. Thus, the formalism shows the connection between contemporary solution approaches and sequence variants in one unified framework. As an example, we apply the formalism to the raised problem of off-resonance artifacts in gradient-spoiled MR Fingerprinting (FISP-MRF).

## Theory

The solution of the homogenized form of the Bloch equations can be obtained by consecutive application of four-dimensional matrix operators (Rotation Operator Algorithm, ROA)^7^, which act on magnetization vectors of the form

2 $${\varvec{M}}({\varvec{r}})=\left(\begin{array}{c}{M}_{+}({\varvec{r}})\\ {M}_{-}({\varvec{r}})\\ {M}_{z}({\varvec{r}})\\ {M}_{0}({\varvec{r}})\end{array}\right),$$

where ${M}_{\pm }={M}_{x}\pm {\text{i}}{M}_{y}$ denotes the transverse magnetization, ${M}_{z}$ the longitudinal magnetization, and ${M}_{0}$ the equilibrium magnetization and ${\varvec{r}}$ is the spatial position with ${\varvec{r}}: = {\left(x,y,z\right)}^{\text{T}}$. Following the nomenclature of Weigel^8^, hard pulses will be denoted by ${T}_{\varphi }\left(\alpha \right)$ with flip angle $\alpha$ and phase $\varphi$. Relaxation and recovery for time $t$ will be written as $R(t)$ implicitly including the dependency on relaxation parameters ${T}_{1}$ and ${T}_{2}$. Off-resonance $\omega$ and gradients ${\varvec{G}}(t)$ lead to phase accrual at position ${\varvec{r}}$ of $\phi =\omega \cdot t+\gamma \int {\text{d}}t {\varvec{G}}(t)\cdot {\varvec{r}}$, which will be denoted by the operator $S\left(\phi \right)$ and where $\gamma$ is the gyromagnetic ratio (${\gamma }_{{}^{1}H}\approx 2\pi \cdot 42.577 \text{MHz}/\text{T}$).

In the theoretical excursion of this work, we will work with four-dimensional representations of the operators, which are presented in Appendix A. For the numerical implementation, however, it is more efficient to work with the original three-dimensional matrices, since the fourth dimension—taking care of equilibrium magnetization—remains unchanged and only needs to be considered for the recovery of longitudinal magnetization to equilibrium.

### Effective RF pulse matrix and the hard pulse approximation

**Figure 1:**
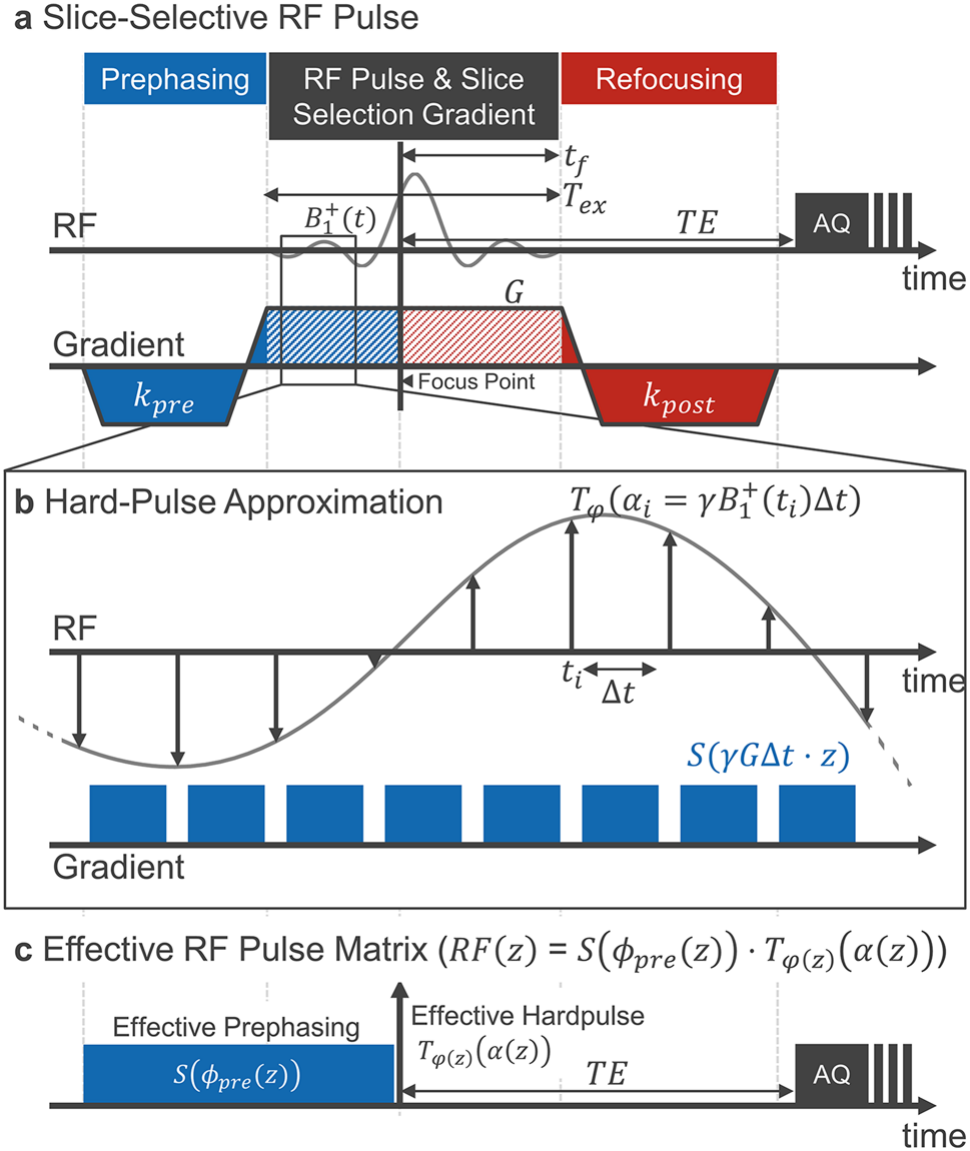
Slice-selective RF pulse and its numerical representation. (**a**) Slice-selection gradient and amplitude-modulated RF pulse for slice-selective excitation shown together with prephasing and refocusing gradients. Same-colored dashed and filled gradient areas are of equal size. (**b**) Graphical representation of the hard pulse approximation assuming short RF pulses compared to rate of change of the B1 + envelope. (**c**) Effective RF pulse representation using the effective slice profile $\alpha (z)$ and pulse phase $\varphi (z)$ as well as an effective prephaser ${\phi }_{\text{pre}}\left(z\right)$.

Slice selection is performed using amplitude-modulated (AM) RF pulses (soft pulse) played concurrently to a constant slice-selection gradient ${\varvec{G}}$ (Fig. 1a)^27^. The through-slice direction is, without loss of generality, defined to be given by $z$, parallel to the slice-select gradient. Pulse duration ${T}_{ex}$, slice selection gradient strength $G$, and the slice thickness $\delta$ are connected by the time-bandwidth product $\kappa$ of the pulse through the relationship^27^

3 $$2\pi \kappa =\gamma G{T}_{ex}\delta .$$

Each RF pulse is preceded by a pre-winder gradient with moment ${k}_{pre}$ and followed by a refocusing gradient of moment ${k}_{post}$. Since the Bloch equations are linear, an effective matrix operator $RF\left(z\right)$ can be defined^28^.

In this work, we restrict the analysis to short pulses (${T}_{ex}\ll {T}_{1},{T}_{2},{T}_{2}^{{\prime}}$) with sufficiently high time-bandwidth product $\kappa$ (${2\pi \kappa \gg T}_{ex}\cdot \omega$), such that relaxation and precession during the pulse can be replaced by relaxation and precession operators acting *after* the pulse. Consequently, the matrix operator acts instantaneously at the “focus point” ${t}_{f}$ of the shaped pulse^29^. The focus point can be obtained through a maximization of the free-induction decay (FID) and is connected to the rewinder area by ${k}_{post}=\gamma G{\cdot t}_{f}$^30^.

The pulse simulation is performed using the hard-pulse approximation (see Fig. 1a,b)^31^. The RF amplitude modulation ${B}_{1}^{+}(t)$ is discretised into hard-pulses of ${T}_{\varphi }\left({\alpha }_{i}\right)$, each acting instantaneously at equidistant time points ${t}_{i}=i\Delta t$ with flip angle ${\alpha }_{i}=\gamma {B}_{1}^{+}\left({t}_{i}\right)\Delta t$. The effective RF pulse matrix is obtained by alternating hard pulses and phase accrual

4 $$RF\left(z\right)=S\left({k}_{post}\cdot z\right)\cdot \left(\prod \limits _{i}S\left(\gamma G\cdot z\boldsymbol{ }\Delta t\right){T}_{\varphi }\left({\alpha }_{i}\right) \right)\cdot S\left({k}_{pre}\cdot z\right).$$

Here, the product ($\Pi$) denotes an ordered matrix product such that earlier time-points are found to the right of later time-points. As relaxation is neglected, the effective RF pulse matrix $RF\left(z\right)$ is a rotation matrix in each $z$ ($\text{det}RF\left(z\right)=1)$. It can be written in the form of an effective hard pulse ${T}_{\varphi \left(z\right)}\left(\alpha \left(z\right)\right)$ and an additional effective phase accrual term $S\left({\phi }_{pre}\left(z\right)\right)$

5 $$RF\left(z\right)={T}_{\varphi \left(z\right)}\left(\alpha \left(z\right)\right)S\left({\phi }_{pre}\left(z\right)\right).$$

$\alpha \left(z\right)$ is the *effective flip angle profile* and $\varphi \left(z\right)$ the *effective pulse phase*, which together determine the *excitation slice profile* of the RF pulse. For echo and storage components, the additional phase-term ${\phi }_{pre}(z)$ needs to be considered (Fig. 1c). This representation is effectively decomposing the RF pulse matrix into Euler angles at each $z$. The resulting intrinsic rotations are first about the z-axis with angle ${\phi }_{pre}\left(z\right)-\varphi (z)$, second, the tipping of the magnetization by the flip angle $\alpha (z)$—a rotation about the rotated ${x}^{{\prime}}$ axis, and finally, a back rotation of $\varphi \left(z\right)$ about the z″-axis.

### Sequence description: MR fingerprinting and configuration state imaging

**Figure 2:**
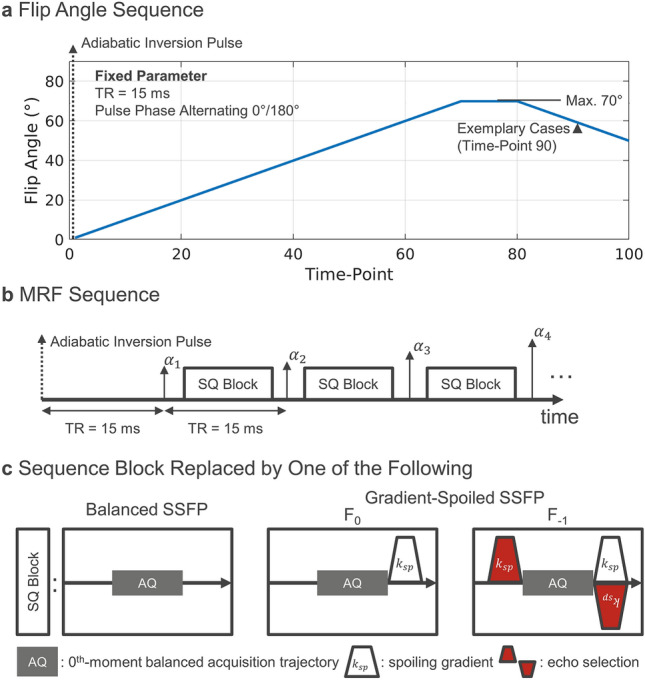
Sequence description. (**a**) Flip angle sequence used for example plots and simulation comparisons. (**b**) Schematic of the employed MRF sequence, where the SQ Block is replaced by one of the blocks shown in (**c**) being either balanced or spoiled SSFP with refocusing of different echo orders using moment-balanced selection gradients (red) played out in spoiling direction.

Throughout this work, we assume constant off-resonance $\omega$, repetition time $TR$ and spoiling gradients with moment ${{\varvec{k}}}_{sp}={{\varvec{e}}}_{z}\cdot {k}_{sp}$ in the direction of slice-selection ${{\varvec{e}}}_{z}$ and an arbitrary set of RF pulses, here given solely by a flip angle variation (Fig. 2a) realized by pulse amplitude scaling.

The fundamental building block of MR Fingerprinting sequences are either balanced or gradient-spoiled SSFP-type sequence modules (Fig. 2b,c)^2,3^. Each is represented by a sequence of operators: slice-selective RF pulse $R{F}_{n}(z)$ with nominal flip angle ${\alpha }_{n}$, where $n$ enumerates consecutive $TR$ intervals, acquisition $AQ$, relaxation $R(TR)$ and phase accrual $S\left(\omega \cdot TR+{k}_{sp}\cdot z\right)$^2,3^. Note, that balanced SSFP is obtained by considering the special case ${{\varvec{k}}}_{sp}=0$.

The magnetization ${{\varvec{M}}}^{(n)}(z)$ directly after the nth RF pulse is then given by

6 $${{\varvec{M}}}^{\left(n\right)}\left(z\right)=R{F}_{n}\left(z\right)\cdot R\left(TR\right)\cdot S\left(\omega \cdot TR+{k}_{sp}\cdot z\right){{\varvec{M}}}^{\left(n-1\right)}\left(z\right),$$

where ${{\varvec{M}}}^{\left(0\right)}\left(z\right)={M}_{0}\left(z\right){\left({0,0},{1,1}\right)}^{T}$ is the initial magnetization. For simplicity, we will assume acquisition to happen instantaneously at the echo time $TE$ relative to the focus point of the soft pulse and assume readout gradients to be zeroth-moment nulled, i.e. magnetization remains unchanged by acquisition. At $TE$, magnetization is given by

7 $${{\varvec{M}}}^{\left(n\right)}\left(z;TE\right)=R\left(TE\right)\cdot {S\left(\omega \cdot TE\right)\cdot {\varvec{M}}}^{\left(n\right)}(z).$$

For spoiled SSFP, configurations other than the FID can be refocused, which is the basis for e.g. double- and triple-echo steady-state (DESS and TESS) relaxometry^32,33^, time-reversed FISP (PSIF or T2-weighted FFE), and diffusion-weighted SSFP sequences^34,35^. Acquisition is preceded by a refocusing gradient with a zeroth moment of integer multiples of ${{\varvec{k}}}_{sp}$. To keep the total spoiling moment constant, compensation gradients with inverse gradient sign are inserted after the readout (Fig. 2c). The magnetization of the *q*th echo is given by

8 $${{\varvec{M}}}_{q}^{\left(n\right)}\left(z;TE\right)=R\left(TE\right){S\left({-q\cdot k}_{sp}\cdot z+\omega \cdot TE\right){\varvec{M}}}^{\left(n\right)}(z).$$

### Hybrid Bloch-EPG formalism

#### Magnetization dynamics

The aim is to reformulate the recurrence Eq. (6) to obtain an analytical expression in the phase accrual $\phi := \omega \cdot TR+{k}_{sp}\cdot z$ per $TR$. This can be achieved, as shown in Appendix B, by a Fourier series expansion of the phase accrual operator

9 $$S\left(\phi \right): = {\text{diag}} \left({e}^{{\text{i}}\phi },{e}^{-{\text{i}}\phi },{1,1}\right) =\sum \limits_{k=-\infty }^{\infty }{e}^{{\text{i}}k\phi}\text{diag}\left({\updelta }_{k,1},{\updelta }_{k,-1},{\updelta }_{k,0}{,\updelta }_{k,0}\right),$$

where ${\delta }_{k,{k}^{{\prime}}}$ is the Kronecker delta (${\delta }_{k,{k}^{{\prime}}}=\{1 :k={k}^{{\prime}};0 :otherwise\}$). The magnetization after the $n$th RF pulse can then be written in the form

10 $${{\varvec{M}}}^{\left(n\right)}\left(z\right)={M}_{0}\left(z\right)\sum \limits_{k}{e}^{{\text{i}}k\left({k}_{sp}\cdot z+\omega \cdot TR\right)}{\cdot {\varvec{x}}}_{k}^{\left(n\right)}\left(z\right),$$

where the Fourier coefficients ${{\varvec{x}}}_{k}^{\left(n\right)}\left(z\right)$ are given by

11 $${{\varvec{x}}}_{k}^{\left(n\right)}\left(z\right)=R{F}_{n}\left(z\right)R\left(TR\right)\cdot\sum\limits_{{k}^{{\prime}}}{\text{diag}}\left({\updelta }_{k,{k}^{{\prime}}+1},{\updelta }_{k,{k}^{{\prime}}-1},{\updelta }_{k,{k}^{{\prime}}}{,\updelta }_{k,{k}^{{\prime}}}\right){{\varvec{x}}}_{{k}^{{\prime}}}^{\left(n-1\right)}\left(z\right).$$

The initial condition translates to ${{\varvec{x}}}_{k}^{\left(0\right)}(z)={\left({0,0},{\delta }_{k,0},{\delta }_{k,0}\right)}^{\text{T}}.$

Through the transformation, the dependency of the magnetization ${{\varvec{M}}}^{\left(n\right)}\left(z\right)$ on the spoiling moment ${k}_{sp}$ and the off-resonance $\omega$ is now fully analytical and given by a Fourier series. The series coefficients ${{\varvec{x}}}_{k}^{\left(n\right)}(z)$ are nothing but continuously spatially-resolved configuration states and we may define ${\left({F}_{k}^{+(n)}\left(z\right),{F}_{k}^{-(n)}\left(z\right),{Z}_{k}^{(n)}\left(z\right),{\delta }_{k,0}\right)}^{T}: = {{\varvec{x}}}_{k}^{\left(n\right)}\left(z\right)$ to relate it to the SR-EPG by Malik et al.^12^ or define $l: = k$ to relate it with the Fourier coefficients ${f}_{l}\left(n\right): = {F}_{k}^{+(n)}$ used in Sobol and Gauntt, which are implicitly dependent on spatial position^26^.

The term ${\sum }_{{k}^{{\prime}}}{\text{diag}}\left({\updelta }_{k,{k}^{{\prime}}+1},{\updelta }_{k,{k}^{{\prime}}-1},{\updelta }_{k,{k}^{{\prime}}}{,\updelta }_{k,{k}^{{\prime}}}\right){{\varvec{x}}}_{{k}^{{\prime}}}^{\left(n-1\right)}$ is a convenient way to write the shifting operator of the EPG in analytical form. The first element of the diagonal matrix corresponds to up-shifting of ${F}^{+}$ and the second to down-shifting of ${F}^{-}$ configurations. $k$ is referred to as the order of the configuration ${{\varvec{x}}}_{k}^{\left(n\right)}(z)$. Here, spatial variation in slice-direction is introduced in the SR-EPG only through the effective RF pulse matrix $R{F}_{n}(z)$, however, variations could also be introduced through spatially-dependent relaxation parameters and transmit-field inhomogenieties^12^.

#### Signal reception

Using the coil sensitivity in slice direction $C\left(z\right)$, which collates all dependencies on coil geometry and induction physics into one convenient sensitivity factor, the signal received directly after the $n$th RF pulse can be written as

12 $${s}^{(n)} ={\int }_{\Omega } {\text{d}}z \, C\left(z\right){M}_{+}^{(n)}(z).$$

The solution ansatz for ${{\varvec{M}}}^{\left(n\right)}\left(z\right)$ (Eq. 10) can be inserted and simplified leading to

13 $${s}^{\left(n\right)}= \sum \limits_{k}{e}^{{\text{i}}k\omega TR}{\int }_{\Omega } {\text{d}}z \, {e}^{{\text{i}}k{k}_{sp}\cdot z}C\left(z\right) \, {M}_{0}\left(z\right) \, { F}_{k}^{+\left(n\right)}\left(z\right).$$

Following Leupold^36^, the signal Eq. (13) can further be extended to include signal decay due to Lorentzian-distributed, microscopic inhomogeneities in the magnetic field distribution leading to an effective decay rate ${R}_{2}^{{\prime}}$, as well as, signal decay and phase accrual to an arbitrary echo time-point $0\le TE\le TR$. This leads us to the hybrid Bloch-EPG equation stated in the introduction

14 $${s}^{\left(n\right)}=\sum \limits_{k}\underset{={W}_{k}\left(\omega , {R}_{2}^{{\prime}},TE\right)}{\underbrace {{e}^{{\text{i}}\omega \left(k\cdot TR+TE\right)-{R}_{2}^{{\prime}}\left|k\cdot TR+TE\right|-\frac{TE}{T2}}}} {\int }_{\Omega } {\text{d}}z \, {e}^{{\text{i}}k{k}_{sp}\cdot z} C\left(z\right) \, {M}_{0}(z) \, {F}_{k}^{+\left(n\right)}\left(z\right).$$

Thus, the acquired signal ${s}^{(n)}$ is determined by summation over configuration orders of the Fourier transformation ${\int }_{\Omega } {\text{d}} z {e}^{ {\text{i}} k{k}_{sp}\cdot z}\cdots$ of *weighted spatially-resolved configurations* ${W}_{k}\left(\omega , {R}_{2}^{{\prime}},TE\right){F}_{k}^{+\left(n\right)}\left(z\right)$. The weighting function ${W}_{k}$ provides the analytical dependency on off-resonance $\omega$, ${R}_{2}^{{\prime}}$ and the $TE$, while the Fourier transformation allows for arbitrary spoiling moments.

Note: Substituting ${k}_{sp}\to {k}_{sp}+\gamma\Delta {B}_{0}({z}_{i})$, the formalism equivalently applies in locally first-order to inhomogeneities in the static magnetic field $\Delta {B}_{0}({z}_{i})$, i.e. local field gradients^26^.

We termed the formalism the *hybrid Bloch-EPG* since it solves the interplay of slice profiles, spoiling gradients, and off-resonance by combining the natural representation of both: (1) extended phase graphs for spoiling and off-resonance and (2) the spatial domain representation of slice profiles as in conventional Bloch simulations.

#### Discretization and spatially-resolved extended phase graphs

For computer simulations, we discretise the spatial domain $\Omega$ in slice direction into $N$ equidistant bins centered at position ${z}_{i}$ with width $\Delta z$ ($i=1\dots N$). We define an interpolation kernel function $I({z}_{i},z)$, which allows us to approximate the RF pulse matrix by

15 $$RF\left(z\right)\approx \sum \limits_{i}RF({z}_{i})\cdot I\left({z}_{i},z\right).$$

For simplicity, we assume the matrix to be *piecewise constant* within each bin leading to the following kernel function

16 $$I\left(z,z{^{\prime}}\right)=\theta \left({z}^{{\prime}}-z+\frac{\Delta z}{2}\right)\theta \left(z-z {^{\prime}}+\frac{\Delta z}{2}\right),$$

where $\theta (\cdot )$ denotes the Heaviside theta function.

Since the configuration states ${{\varvec{x}}}_{k}^{(n)}(z)$ only depend on $z$ via $RF(z)$, the ${{\varvec{x}}}_{k}^{\left(n\right)}\left(z\right)$ are also piecewise constant and can be written as

17 $${{\varvec{x}}}_{k}^{\left(n\right)}\left(z\right)\approx \sum \limits_{i}{{\varvec{x}}}_{k,i}^{\left(n\right)}\cdot I\left({z}_{i},z\right).$$

Insertion into the continuous hybrid Bloch-EPG Eq. (14), making use of the Fourier transformation ${\mathcal{F}}_{k,i}({k}_{sp})$ of the kernel $I{(z}_{i},z)$

18 $${\mathcal{F}}_{k,i}\left({k}_{sp}\right): = {\int }_{\Omega }dz \, I\left({z}_{i},z\right){e}^{ik{k}_{sp}\cdot z}=\Delta z \, {\text{sinc}}\left(k\frac{{k}_{sp}\Delta z}{2}\right){e}^{ik{{k}_{sp}\cdot z}_{i}}$$

identifying the discrete spatially-resolved configuration states ${\left({F}_{k,i}^{+(n)},{F}_{k,i}^{-(n)},{Z}_{k,i}^{(n)},{\delta }_{k,0}\right)}^{T}: = {{\varvec{x}}}_{k,i}^{\left(n\right)}$, and assuming spatially constant coil sensitivity $C$ and equilibrium magnetization ${M}_{0}$, we arrive at the approximate solution to the hybrid Bloch-EPG

19 $${s}^{\left(n\right)}\approx C{M}_{0}\sum \limits_{k}{W}_{k}\left(\omega , {R}_{2}^{{\prime}},TE\right) \sum \limits_{i}{\mathcal{F}}_{k,i}({k}_{sp}) \, {F}_{k,i}^{+\left(n\right)},$$

which can be directly evaluated from a spatially-resolved extended phase graph simulation. The term $\Delta z {\text{sinc}}\left(k\frac{{k}_{sp}\Delta z}{2}\right)$ in the kernel’s Fourier transformation ${\mathcal{F}}_{k,i}\left({k}_{sp}\right)$ is equivalent to the “damping factor” ${D}_{l}$ introduced in Sobol and Gauntt^26^, and accounts for dephasing within each bin (voxel). Note: the equivalent formulation derived by Sobol and Gauntt was stated for a single voxel, i.e. without the summation over the slice profile denoted by ${\sum }_{i}\cdots$ here.

#### Configuration state imaging

For refocusing of an arbitrary configuration $q$—in the following we will call it the *refocused echo* $q$ to distinguish it from *configuration state* ${F}_{k}^{+}$—we can modify Eq. (19) accordingly

20 $${s}_{q}^{\left(n\right)}\approx C{M}_{0} \sum \limits_{k}{W}_{k}\left(\omega , {R}_{2}^{{\prime}},TE\right)\sum \limits_{i}{\mathcal{F}}_{k-q,i}({k}_{sp}) \, {F}_{k,i}^{+\left(n\right)}.$$

### Connection to Bloch and slice-selective EPG simulations

The hybrid Bloch-EPG formulation can be used to obtain Bloch and slice-selective EPG representations for the same sequence. Direct evaluation of the transverse magnetization ${M}_{+}^{\left(n\right)}(z)$ at the discrete positions ${z}_{i}$ before spatial integration, gives access to the solution obtained through conventional Bloch simulation, i.e. the time-dependent spatial magnetization distribution

21 $${M}_{+}^{\left(n\right)}\left({z}_{i}\right)={M}_{0} \sum \limits_{k}{W}_{k}\left(\omega , {R}_{2}^{{\prime}},TE\right){e}^{{\text{i}}\left(k-q\right){k}_{sp}\cdot z} \sum \limits_{i}{F}_{k,i}^{+\left(n\right)}.$$

On the other hand, Fourier transformation of the transverse magnetization allows to recover the solution of the slice-selective EPG (ssEPG), which directly yields the magnetization’s k-space representation by performing the hard pulse approximation using the EPG (here, to distinguish configuration order $k$ from k-space, $k{^{\prime}}$ is used to denote spatial frequencies)

22 $${F}_{+}^{(n)}\left({k}^{{\prime}}\right)={M}_{0}\Delta z \sum \limits_{k}{W}_{k}\left(\omega , {R}_{2}^{{\prime}},TE\right) \sum \limits_{i}{e}^{{\text{i}}\left((k-q) {k}_{sp}-{k}^{{\prime}}\right)\cdot {z}_{i}}\cdot {\text{sinc}}\left((k-q)\frac{{k}_{sp}\Delta z}{2}\right) {F}_{k,i}^{+\left(n\right)}.$$

**Figure 3:**
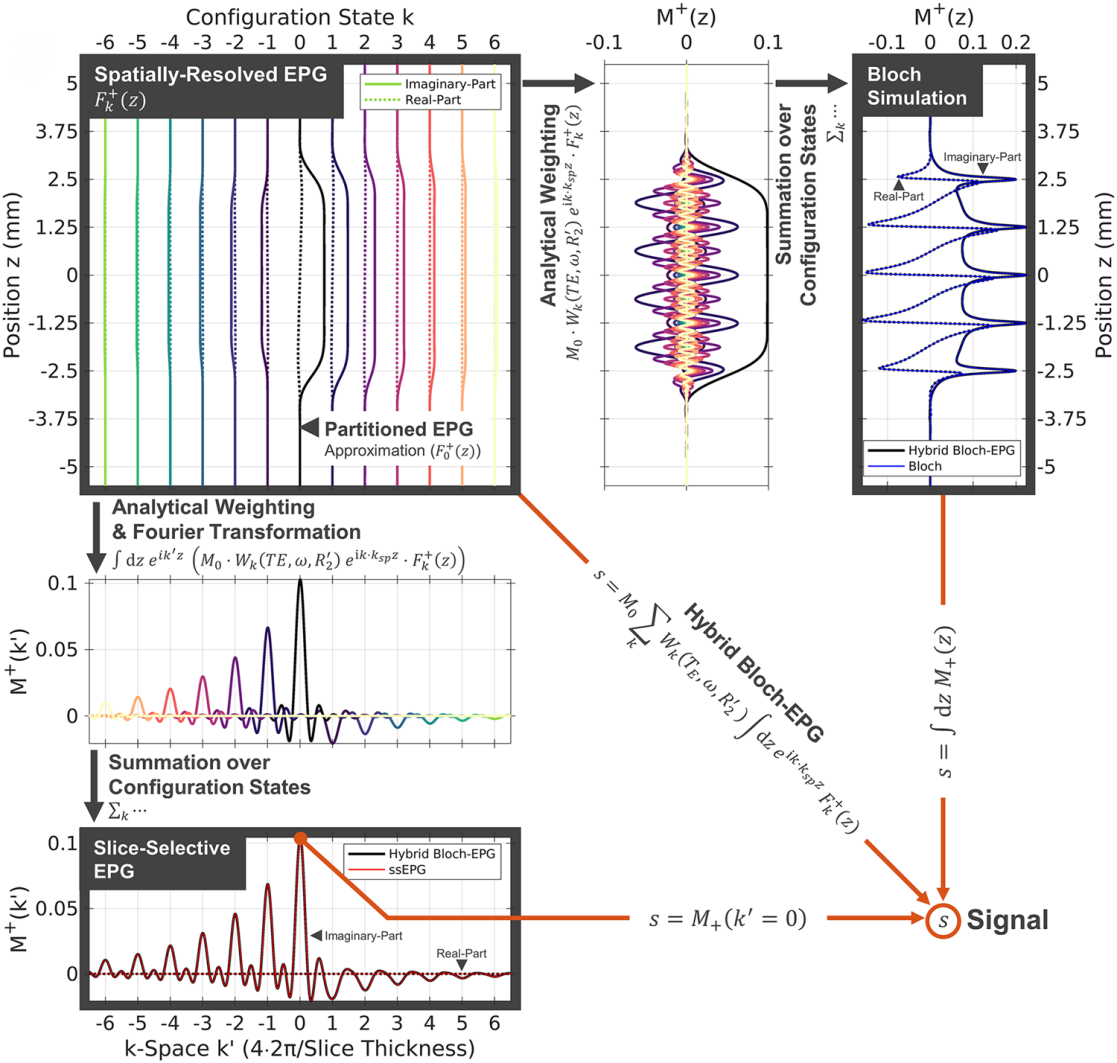
Graphical Representation and Relationship of Hybrid Bloch-EPG, Spatially-Resolved EPG, Partitioned EPG, Bloch Simulation, and Slice-Selective EPG. Bloch simulation (top, right) and slice-selective EPG (bottom, left) are equivalent up to Fourier transformation. Using the spatially-resolved EPG (SR-EPG; top, left) and the hybrid Bloch-EPG formalism, both Bloch and ssEPG solutions can be obtained using an analytical relationship. The intermediate steps show the transformation and weighting of each configuration state separately for both the spatial domain (top) and k-space (left). Through summation over state orders $k$, interference between configuration states leads to modification of the slice profile and hence a change in observed signal. A single SR-EPG simulation provides analytical access to four dimensions of the solution space: *TE*, $\omega$, ${R}_{2}^{{\prime}}$, and the spoiling moment ${k}_{sp}$. While ssEPG and Bloch simulations can be obtained from each other, the mapping of SR-EPG to Bloch or ssEPG cannot be reversed as the configurations cannot be disentangled from the spatial information alone. The partitioned EPG (pEPG) approximation is given by summation over the F0 state in the SR-EPG (top, left). Solid and broken lines denote imaginary- and real-part, respectively.

These relationships are graphically depicted in Fig. 3. A spatially-resolved EPG is shown in the top left corner for the configuration order $k=-6\dots 6$ as a function of the position $z$ along the slice profile.

#### Bloch simulation (Fig. 3, left to right)

Analytical weighting with the weighting kernel ${W}_{k}\left(\omega , {R}_{2}^{{\prime}},TE\right)$ as well as resolving the spoiling and echo refocusing by applying ${e}^{{\text{i}}(k-q){k}_{sp}\cdot z}$ to the data is employed to resolve the intermediate representation of the configuration states. These are then superimposed to obtain the slice profile through the hybrid Bloch-EPG formulation (black, right), which is equivalent to direct Bloch simulation (blue). The root-mean-square error of the complex slice profiles normalized to the signal amplitude obtained via Bloch simulation (nRMSE) is 0.71% (see “Methods”, Eq. (40)). By integrating over the magnetization profile, signal ${s}^{(n)}$ is obtained. The predicted MR signal deviates by $3.8\cdot {10}^{-4}\%$ between hybrid Bloch-EPG and Bloch simulation.

Note: A change in off-resonance (here, $\omega =0$ is shown) will lead to different relative phases of the weighted configurations leading to a phase shift of the oscillations in the intermediate representation. This will modify the slice profile by interference and lead to variations in the gradient-echo signal, which has been observed and described for MRF previously^18,22,23^.

#### Slice-selective EPG (Fig. 3, top to bottom)

Similarly, we arrive at the intermediate k-space representation ($k{^{\prime}}$) through spatial Fourier transformation, where configuration states appear to be shifted by the spoiling moment ${k}_{sp}$. Here, off-resonance $\omega$ will modify the complex phase of each configuration. Summing over the configuration order $k$ leads to the slice-selective EPG representation in the bottom row, where the weighted configurations will interfere, which was previously described by Sobol and Gauntt^26^. The nRMSE of the k-spaces of hybrid Bloch-EPG and ssEPG is 0.15% (see “Methods”, Eq. (41)). Here, the MR signal is given by the DC component (${k}^{{\prime}}=0$) as known from conventional EPGs^8,12,18^. The predicted signal of the ssEPG deviates from the Bloch simulation by $2\cdot {10}^{-2}\%$. Refocusing of higher configuration orders leads to shifting of the state picture, centering a different peak at $k^{\prime}=0$ and the formation of the $q$th refocused echo.

### Special cases of spoiling: balanced SSFP and partitioned EPG

Two special cases of spoiling can be discussed, ${k}_{sp}=0$ equivalent to balanced SSFP; and ${k}_{sp}\to \infty$, which will be shown to be equivalent to the partitioned EPG solution.

#### ${k}_{sp}=0$—balanced SSFP

Balanced SSFP can be considered a special case of spoiled sequences with vanishing spoiling moment (${k}_{sp}=0$). In this case, the hybrid Bloch-EPG Eq. (20) can be simplified to

23 $${s}_{bSSFP}^{\left(n\right)}=C{M}_{0}\Delta z \sum \limits_{k}{W}_{k}\left(\omega , {R}_{2}^{{\prime}},TE\right) \sum \limits_{i} {F}_{k,i}^{+\left(n\right)}.$$

Hence, in balanced SSFP, the configuration states only contain information about off-resonance, which is by definition independent of the spatial coordinate. Thus, slice profiles can directly be integrated over before weighting and summation.

#### ${k}_{sp}\to \infty$—partitioned EPG

In the limit ${k}_{sp}\to \infty$, the sinc-function in Eq. (18) can be simplified to^37^

24 $$\underset{{k}_{sp}\to \infty }{\text{lim}} {\text{sinc}} \left(k\frac{{k}_{sp}\Delta z}{2}\right)={\delta }_{k,0},$$

leading Eq. (20) to become

25 $${s}_{q,pEPG}^{\left(n\right)}=\underset{{k}_{\mathit{sp}}\to \infty }{\text{lim}}{s}_{q}^{\left(n\right)}={CM}_{0}\Delta z\cdot {W}_{q}\left(\omega , {R}_{2}^{{\prime}},TE\right) \sum \limits_{i} {F}_{q,i}^{+\left(n\right)}.$$

Hence in the limit of infinite spoiling, the slice profile integration can be directly performed and only *one state,* the $q$-th configuration will contribute. Interference of adjacent configurations, as predicted by the hybrid Bloch-EPG, does not take place. This implies that no magnitude or phase variation other than the trivial phase accrual $\omega \cdot (qTR+TE)$ can be observed^18^. This approximation is equivalent to the approach by Lebel and McPhee, which was termed *partitioned EPG (pEPG)* by Ostenson^13,14,18^.

**Figure 4:**
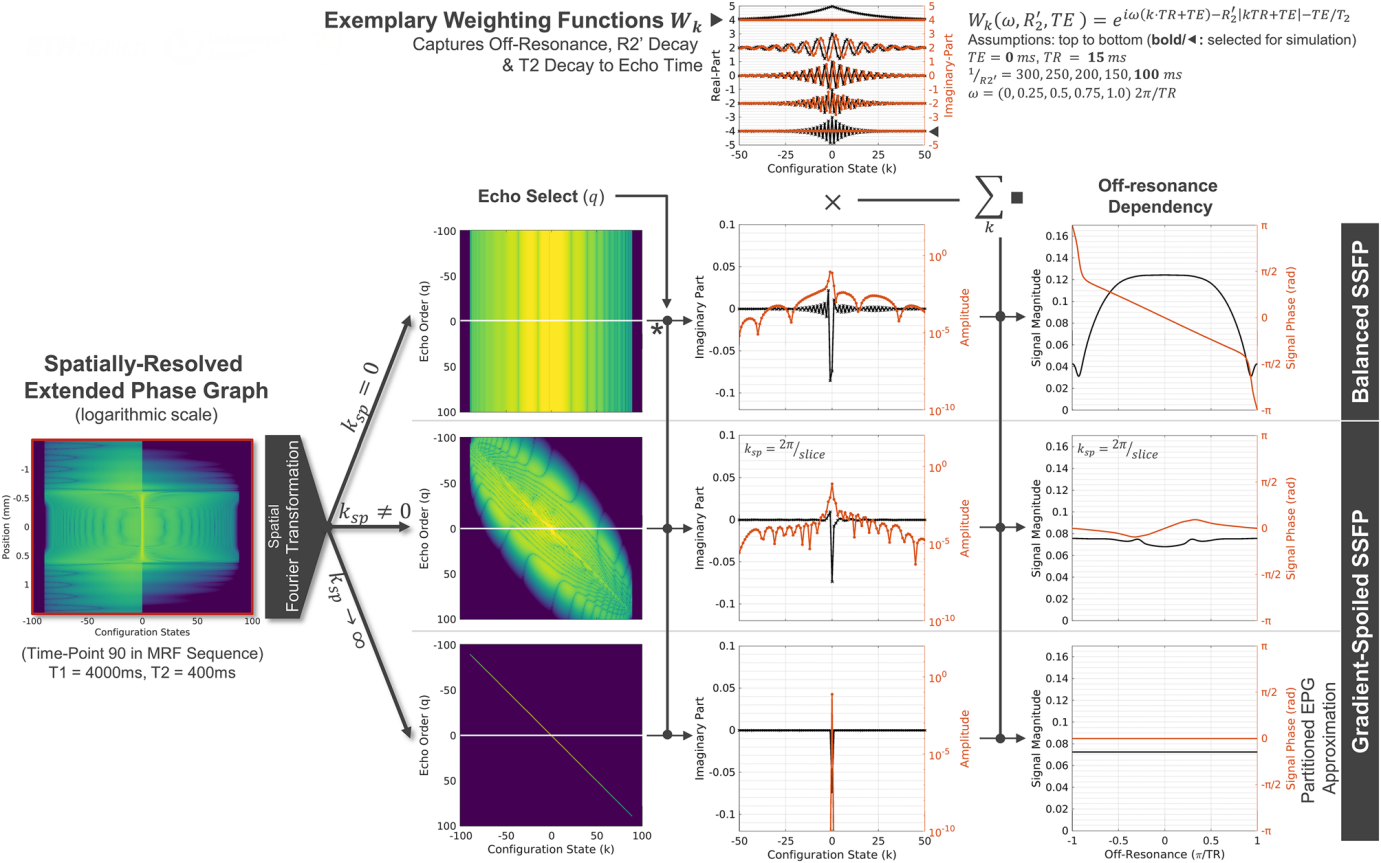
Analytical recovery of balanced SSFP, spoiled SSFP and pEPG from one spatially-resolved phase graph. Visualization of the three spoiling cases which can be analytically recovered from a single spatially-resolved EPG (SR-EPG) simulation by applying the Fourier transformation, echo selection (white line, here the 0th order), and configuration state weighting ${W}_{k}.$ Three spoiling cases can be identified: (1) no gradient spoiling is equivalent to balanced SSFP, where the interference of configuration states leads to the distinct frequency response profile. Here, the signal is degenerate in the echo order (*). (2) With finite spoiling, banding artifacts are reduced at the expense of overall signal magnitude. However, as already noted by Ostenson et al.^18^, an off-resonance dependency remains, which is sensitive to the spoiling moment. (3) For infinite spoiling, a single state will contribute the signal, which is equivalent to the solution of the partitioned EPG (pEPG). Here, banding is fully suppressed and no off-resonance dependency apart from the trivial phase accrual at TE is observed. For the simulation of the off-resonance dependency, the employed weighting function is marked and the accompanying parameters are given in bold.

Figure 4 visualizes the signal formation within the hybrid Bloch-EPG from one SR-EPG (left) for the three possible cases of spoiling: no spoiling (${k}_{sp}=0$, top row), finite spoiling (${k}_{sp}\ne 0$, middle row) and strong spoiling (${k}_{sp}\to \infty$, bottom row). After spatial Fourier transformation, a configuration order $k$ vs. refocused echo $q$ plot can be obtained (left column). For any given $q$, configurations along the white, horizontal lines are selected. With ${k}_{sp}=0$, the echo order $q$ is irrelevant as no spoiling moment is applied, i.e. it leads to degeneracy for all echo orders. For ${k}_{sp}\to \infty$ (bottom row), only one configuration remains, which leads to the pEPG solution. Configurations are weighted by ${W}_{k}$ (top), which contributes the analytical dependency on $TE$, ${R}_{2}^{{\prime}}$ and $\omega$. Summation over the weighted configurations allows to recover, e.g. the signal dependency on off-resonance, here shown for $TE=0$.

**Figure 5:**
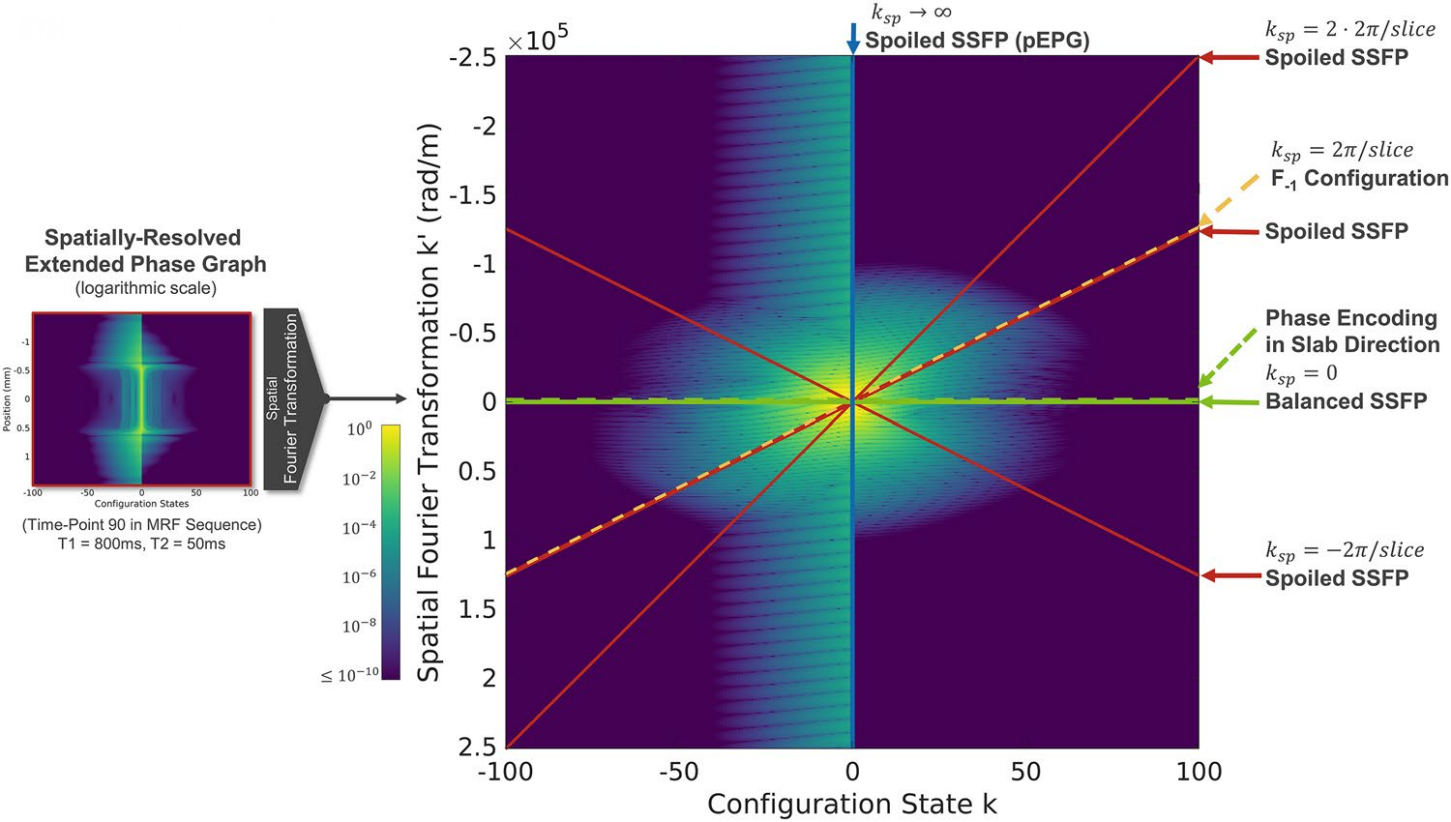
Interpretation of balanced and spoiled SSFP in the configuration state k vs. k-space (k’) picture. Application of the Fourier transformation and integration over the slice profile is equivalent to summing along lines in the configuration state k vs. k-space k’ plot. Horizontal lines (green) are attributed to balanced SSFP, where a shift away from k-space center can be interpreted as phase encoding in slab direction (broken line, vertically offset). Angulated lines correspond to spoiled SSFP. Here, shifts $\ll {\text{k}}_{\text{sp}}$ denote phase encoding, whereas shifts on the order or more of the spoiling moment can be attributed to refocusing of higher configuration orders (e.g. PSIF or time-reversed FISP sequences; broken, orange line). The pEPG solution is identified with a vertical line of infinite slope (blue).

To further the understanding of the three cases of spoiling in SSFP, Fig. 5 shows the possible gradient echo sequences in a combined configuration k vs. k-space ($k {^{\prime}}$) graph. The plot on the right is achieved by spatially Fourier transforming the SR-EPG on the left. During signal acquisition, a superposition of weighted configuration states ${\sum }_{k}{W}_{k}{F}_{k}^{+}$ is observed. Each configuration state is evaluated at a different spatial frequency $k^{\prime}$, which can be found by selecting them along straight lines.

Balanced SSFP corresponds to selecting spatial frequencies of configurations along horizontal lines (green), i.e. all configurations are evaluated at the same spatial frequency. Angulated lines (red) depict spoiled SSFP sequences. The slope is given by the spoiling moment ${k}_{sp}$. Asymmetry in the slice profile phase (see Fig. 11) arising from the non-linear response of the Bloch equation’s solution with respect to RF fields leads to an asymmetric k′-space, which is seen in Fig. 5 by the asymmetry between the upper and the lower half-space. Hence, negative spoiling moments lead to a different spoiled SSFP signal than positive. Vertical shifting of lines (broken) corresponds to adding gradients before the acquisition object with respective compensation gradients after the readout, i.e. phase encoding gradients. Small shifts ($\ll {k}_{sp}$) are attributed to phase encoding as used in Fourier imaging (green, broken). Shifts of integer multiples of ${k}_{sp}$ refocus different configuration orders, e.g. ${F}_{-1}$ or the PSIF-echo (orange, broken).

## Results

### Application of the hybrid Bloch-EPG to MR fingerprinting and configuration state imaging

#### Off-resonance

**Figure 6:**
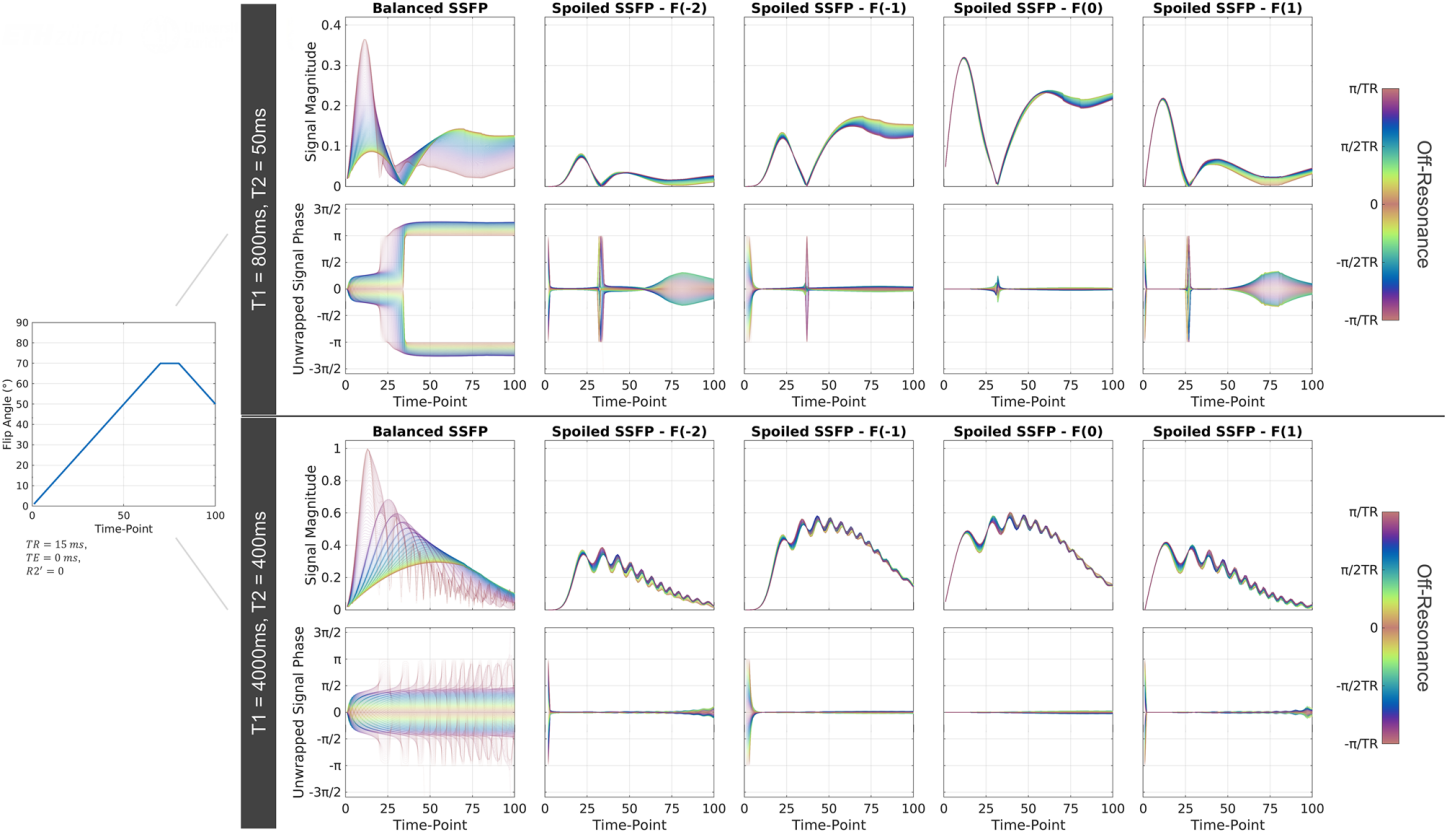
Signal Magnitude and phase for balanced and spoiled SSFP-based MRF sequences as a function of off-resonance. Signal-time curves for 101 off-resonance values between $\pm\uppi /{\text{TR}}$ are shown. Positive and negative frequency offsets share the same color as they are indistinguishable in the signal magnitude response. For spoiled SSFP, the signal phase is shown relative to the phase accrual observed within the pEPG formalism, i.e. accounting for sign change due to inversion recovery and the trivial accrual of $\upomega ({\text{qTR}}+TE)$ due to the refocusing of different configuration orders $q$. Spoiled SSFP (${\text{k}}_{\text{sp}}=4\cdot 2\uppi /\updelta$, $\delta :$ slice thickness) shows greatly reduced off-resonance dependency compared to bSSFP. Higher configuration orders experience larger magnitude and phase variation, which can be attributed to neighbouring, lower configurations being of larger amplitude, thus leading to stronger interference.

In Fig. 6, the signal is plotted as a function of time index and for two different relaxation parameters for both balanced and spoiled SSFP sequences. The respective off-resonance value is color coded, where positive and negative off-resonance share the same color bar as they are indistinguishable in the magnitude plots. The spoiling moment of ${k}_{sp}=4\cdot 2\pi /\delta$ reduces the off-resonance dependency greatly compared to bSSFP for all displayed configuration orders. While magnitude variation persists equally for all configurations, phase variation is increased for higher-order configurations. Phase variation is predominantly found in the region of low signal magnitude as well as for high flip angles.

#### R2′, off-resonance and echo-time dependency

**Figure 7:**
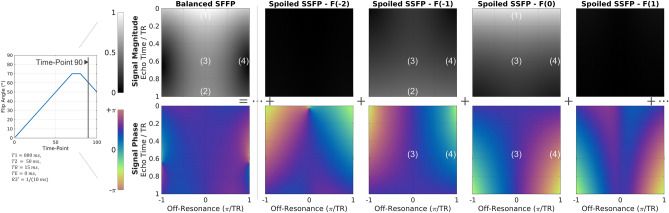
Dependency of signal amplitude and phase on echo time and off-resonance. The bSFFP-MRF signal can be obtained by superimposing the signal of all configuration state orders F(k). Signal-behavior of bSSFP-MRF is dominated by (1) GRE at short echo times and (2) spin-echo or time-reversed GRE for echo times close to TR. For high R2′ decay rates, higher-order contributions can be neglected. Constructive (3) and destructive (4) interference of configuration states of different order lead to the distinctive bSSFP-banding structure.

In Fig. 7, the signal for time-point 90 in the MR fingerprinting sequence is shown for both balanced and spoiled SSFP sequences and four configuration orders. As in conventional balanced SSFP, the bSSFP-MRF signal behaves like a gradient-echo for short TE (1), and spin-echo like for long TE (2)^38^. Configurations of higher order (${F}_{1}$ and ${F}_{-2}$) cannot be observed due to the rapid transverse decay for R2′ = 1/10 ms. Constructive (3) and destructive (4) interference of configurations leads to the banding structure observed in balanced SSFP.

#### Spoiling moment

**Figure 8:**
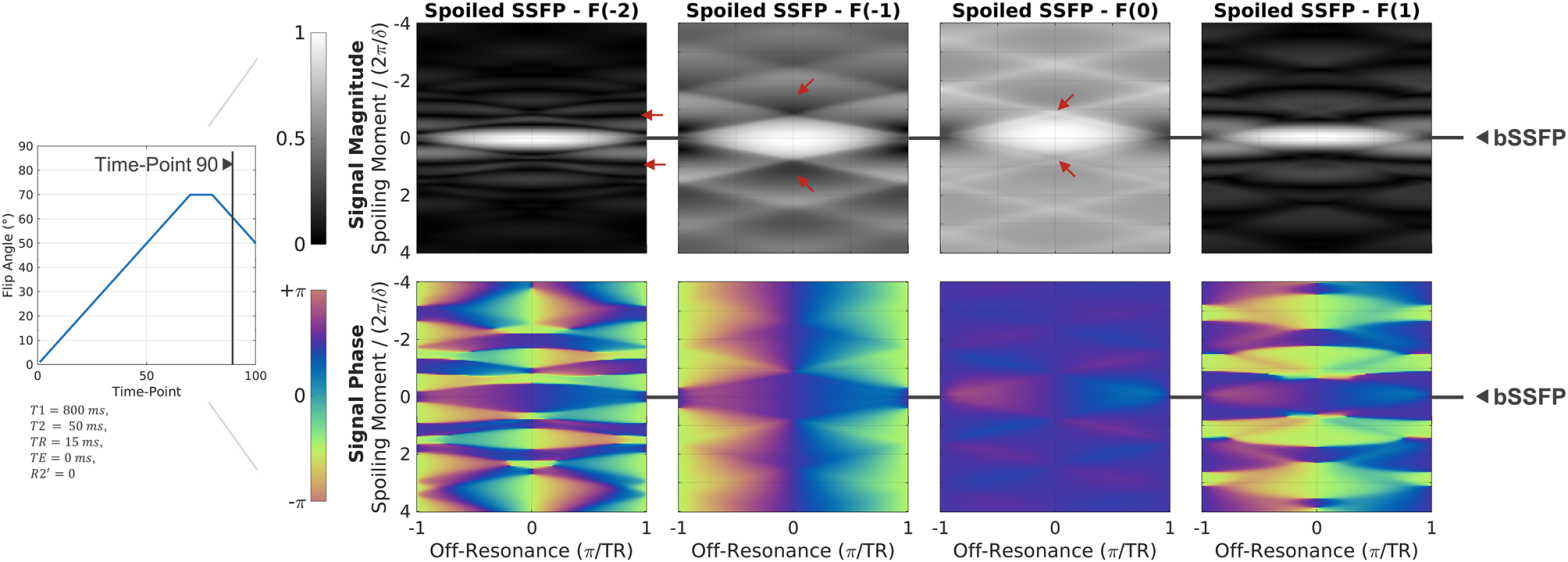
Dependency of the spoiled SSFP signal on off-resonance and spoiling moment. At ${\text{k}}_{\text{sp}}=0$, the balanced SSFP signal is recovered for all cases. The asymmetry in the slice profile phase leads to different responses for positive and negative spoiling (red arrows). For four-fold spoiling, variation in signal amplitude and non-linear variation in phase is mostly suppressed.

In Fig. 8, the signal response for time-point 90 is plotted against off-resonance and spoiling moment for the four configuration orders. At ${k}_{sp}=0$, all configurations show the same balanced SSFP response. For small deviations of ${k}_{sp}$ from zero, the different configuration states can be thought of as phase encodings of the balanced SSFP slice profiles. Asymmetry in the effective slice profile leads to different response for positive and negative spoiling moments. For sufficiently large spoiling moments, both magnitude and non-linear phase variations can be effectively suppressed.

#### Suppression of magnitude variation in MRF

**Figure 9:**
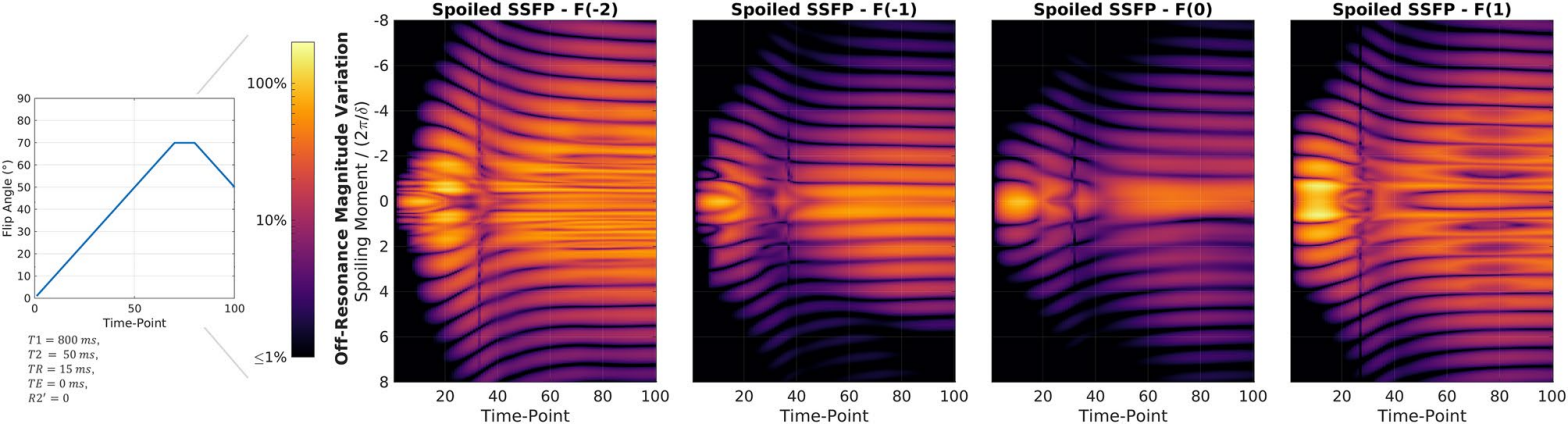
Off-resonance signal variation as a function of time point and spoiling moment. standard deviation of the signal magnitude with respect to off-resonance normalized to the mean signal amplitude plotted against spoiling moment and temporal index. Between index 15 and 50, minima of the signal variation do not follow a horizontal line, thus, no single spoiling moment can be found that reduces slice profile induced off-resonance variation to below 1% in this exemplary case. Only with strong spoiling ($\ge 8\cdot 2\uppi /\updelta$) can the variation consistently be suppressed for the F0 configuration. This demonstrates that the pEPG is ill-suited to predict MRF signal time-courses for standard spoiled SSFP-based MRF and even more so in the context of configuration state imaging.

Signal magnitude and phase vary with spoiling moment and fingerprinting index. Figure 9 shows the standard deviation of the signal magnitude with respect to off-resonance normalized to the average MRF signal

26 $$\frac{\sqrt{{\left \langle {\left(\left|{S}^{\left(n\right)}\left({k}_{sp},\omega \right)\right|- { \left \langle \left|{S}^{\left(n\right)}\left({k}_{sp},\omega \right)\right| \right \rangle }_{\omega }\right)}^{2} \right \rangle }_{\omega }}}{{\left \langle \left|{S}^{\left(n\right)}\left({k}_{sp},\omega \right)\right| \right \rangle }_{\omega ,n}}$$

as a function of spoiling moment ${k}_{sp}$ and time $n$, where ${\langle \cdots \rangle }_{\omega }$ denotes the average over $\omega$. Periodic minima are observed, which are encountered at approximately $2\pi /\delta$. In the time-point region between 15 and 50 (flip angle ramp between 15° and 50°), the location of the minima varies. Thus, no spoiling moment can be found that reduces the off-resonance signal variation below 1% (black) for all time points simultaneously. For gradient-recalled echo (GRE, ${F}_{0}$ state of gradient-spoiled SSFP) and time-reversed GRE (${F}_{-1}$ state of gradient-spoiled SSFP), a spoiling moment of at least $8\cdot 2\pi /\delta$ is necessary to reduce magnitude variation below 1%.

### The role of the damping factor: aliasing and Bloch simulations

**Figure 10:**
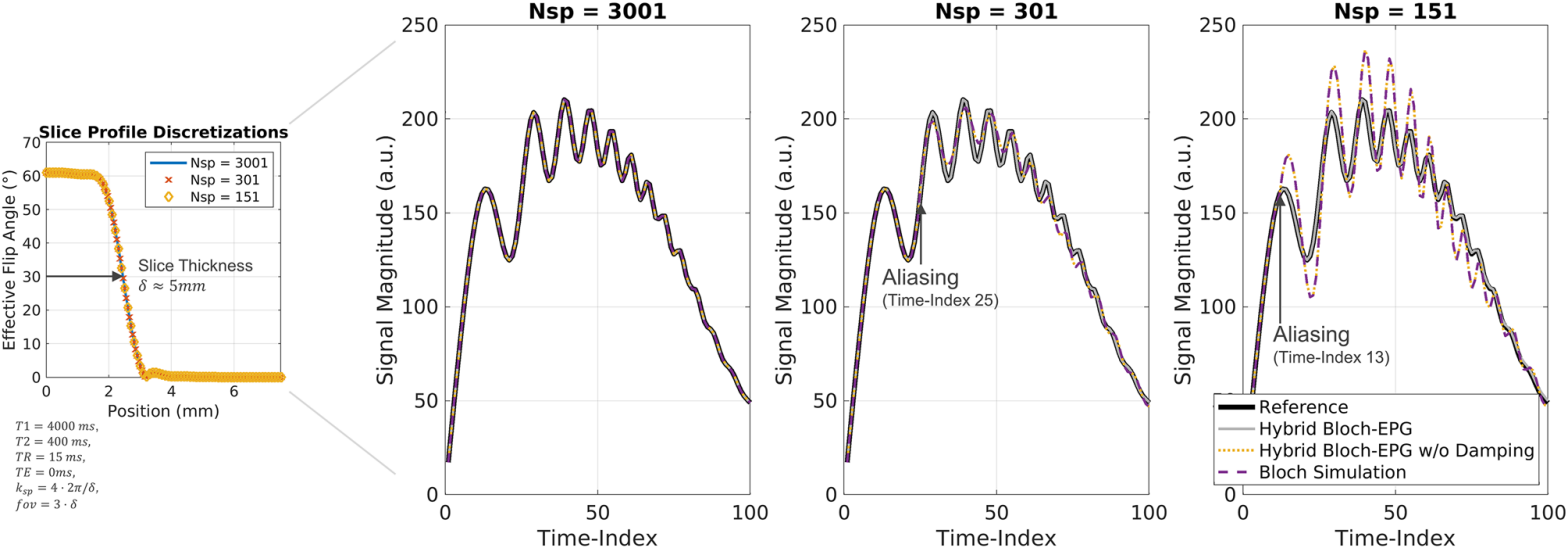
Comparison of signal-time behavior for different slice profile discretizations and simulation methods. The hybrid Bloch-EPG with reduced number of slice profile discretization points ${N}_{sp}$ (solid, gray) approximates well the signal obtained with the Hybrid Bloch-EPG and ${N}_{sp}=3001$ (black). Omitting the damping factor in the hybrid Bloch-EPG signal Eq. (19) leads to aliasing (yellow, dotted), which is equivalent to the results of a Bloch simulation (violet, broken).

In Fig. 10, the predicted signal behavior is compared between hybrid Bloch-EPG and a standard Bloch simulation for different number of slice profile discretization points ${N}_{sp}$. The hybrid Bloch-EPG with a reduced number of discretization points (gray) produces comparable results to the reference calculation with 3001 points (black). In contrast, the result of the Bloch simulation (violet, broken) shows aliasing for both 301 and 151 points. Equivalent aliased results are obtained when omitting the *damping factor* ${\text{sinc}}\left(k\frac{{k}_{sp}\Delta z}{2}\right)$ in Eq. (18) in the hybrid Bloch-EPG (yellow, dotted).

This demonstrates the importance of the damping factor to resolve the effect of spoiling on a sub-discretization level. Aliasing occurs as soon as the damping factor becomes zero for the highest occupied state ${k}_{max}$

27 $${\text{sinc}}\left({k}_{max}\frac{{k}_{sp}\Delta z}{2}\right)=0,$$

i.e. a state ${k}_{max}$ is populated, which can no longer be resolved by the slice profile discretization alone. The respective configuration state at which aliasing occurs is given by

28 $${k}_{max}=\frac{2\pi }{\Delta z\cdot {k}_{sp}}$$

and evaluates to 250, 25 and 12.6 for the shown case, which is in agreement with the findings shown in Fig. 10.

## Discussion

We have presented a formalism that employs the spatially-resolved EPG as an input to obtain the MR signal of spoiled and balanced SSFP sequences with variable, slice-selective RF pulses. The formalism allows to capture the dependency on spoiling moment ${k}_{sp}$, echo time $TE$, microscopic dephasing $R2^{\prime}$, and off-resonance $\omega$ analytically. We recovered three known solution methods to the Bloch equations, i.e. spatial-domain simulations (Bloch simulation), slice-selective extended phase graphs (ssEPG), and the partitioned EPG (pEPG) and presented them in one unified picture.

### Interpretation of the hybrid Bloch-EPG

Considering gradient-spoiled SSFP, spoiling gradients store magnetization history in high spatial frequencies of the magnetization distribution. The present formalism suggests that off-resonance artifacts in spoiled SSFP sequences arise from overlapping *past* and *current* magnetization in k-space leading to interference of configuration states. This occurs whenever a state’s k-space representation has finite intensity at multiples of the spoiling moment. Since each configuration order $$k$$ shows different off-resonance phase $$\omega$$($$kTR+TE)$$, interference of the magnetization states leads to signal magnitude and phase variations. Changes in the spoiling moment can either reduce or amplify interference. Generally, the overlap of the configurations is reduced with increasing spoiling moment^26^.

Through the analytical dependency on the spoiling moment, the special case ${k}_{sp}=0$ can be considered, which corresponds to balanced SSFP (True-FISP). To the knowledge of the authors, this is the first time that a common formalism for balanced and spoiled SSFP-based MRF sequences is presented that allows to seamlessly and analytically sweep between these sequences. This also applies when fully neglecting any spatial variation in the magnetization apart from spoiling (pEPG). In this case, interference of configuration states does not occur for ${k}_{sp}\ne 0$. However, setting ${k}_{sp}=0$, the balanced SSFP solution can again be obtained^36^. Since SR-EPG calculations are readily implemented and already widely used, adoption of the hybrid Bloch-EPG formula is straightforward.

Fundamental to the slice-selective EPG formalism is a constant spoiling moment increment $\Delta k$, which is given by the time-resolution $\Delta t$ employed in the hard pulse approximation (Eq. 4)

29 $$\Delta k=\gamma G\Delta t.$$

In order to ensure linear phase graph growth, spoiling moment ${k}_{sp}$, refocusing gradient moment ${k}_{post}$ and prewinder ${k}_{pre}$ need to be integer multiples of $\Delta k$. This can either be enforced through rounding, rendering the solution inexact or by designing the pulse sequence respectively. Contrary, in Bloch simulations, the minimal number of isochromats is determined by the spoiling moment and number of sequence repetitions simulated to avoid aliasing artifacts. In the hybrid Bloch-EPG, however, the mathematical separation of slice profile and spoiling means that aliasing cannot occur, and the slice profile resolution can be chosen according to accuracy needs (Fig. 10). Here, the damping factor plays a crucial role by analytically resolving the intra-voxel phase dispersion on the sub-discretization level. Likewise, the number of configuration states solely depends on the number of time points simulated for the MRF sequence and is fully independent of the time-resolution of the RF pulse. This flexibility and intrinsic numerical stability of the method comes at the expense of increased memory requirements, as the full SR-EPG needs to be retained in order to evaluate the analytical dependencies. The implementations of Bloch, ssEPG and hybrid Bloch-EPG presented here were not designed with computational efficiency in mind. An exact computational complexity analysis and performance comparison of the techniques in the realm of MRF dictionary generation is beyond the scope of this work.

### Extensions to the hybrid Bloch-EPG

#### Off-resonance distribution

In Eq. (14), we followed Leupold^36^, to extend our model to include dephasing due to microscopic field inhomogeneities effectively assuming a Lorentzian off-resonance distribution. Using the work of Ganter^39^, other distributions can equally be considered by integrating the signal ${s}^{(n)}$ over an off-resonance distribution $p\left(\omega \right)$. Since $\omega$ only enters into ${s}^{(n)}$ via the exponential term ${e}^{{\text{i}}\omega \left(k\cdot TR+TE\right)}$ contained in the weighting function ${W}_{k}$, we can readily define a generalized weighting function

30 $${W}_{k}\left(\omega , TE;p\left(k\right)\right): = {e}^{-\frac{TE}{T2}}\cdot \tilde{p }\left(k\cdot {T}_{R}+TE\right),$$

where $\tilde{p }\left(t\right)$ is the Fourier transformation of the off-resonance distribution function $p\left(\omega \right)$

31 $$\tilde{p }\left(t\right)=\underset{-\infty }{\overset{\infty }{\int }} {\text{d}} \omega \, p\left(\omega \right){e}^{{\text{i}}\omega t}.$$

For example, by substituting the Fourier transformations provided by Ganter and keeping a global offset frequency $\omega$, we arrive at the following two expressions for the weighting function for Gaussian-distributed inhomogeneities

32 $${W}_{k}^{Gauss}\left(\omega , \sigma ,TE\right)={e}^{{\text{i}}\omega \left(k\cdot TR+TE\right)-\frac{TE}{T2}-\frac{{\sigma }^{2}}{2}{\left(k\cdot TR+TE\right)}^{2}}$$

and spherically distributed inhomogeneities

33 $${W}_{k}^{spherical}\left(\omega , \sigma ,TE\right)={e}^{{\text{i}}\omega \left(k\cdot TR+TE\right)-\frac{TE}{T2}}\frac{{J}_{1}\left(2\sigma \left(k\cdot TR+TE\right)\right)}{\sigma \left(k\cdot TR+TE\right)}$$

with ${J}_{1}$ being the Bessel function of the first kind^37^.

#### Spectral information

Analogously, the model can be extended to include multi-peak spectra analytically, e.g. for accurate modelling of fat using a 7-peak model^40^. Assuming peak frequencies of ${\omega }_{j}$, line-width ${R}_{2,j}^{{\prime}}$, and peak weights ${w}_{j}$, the weighting function can be replaced by

34 $${W}_{k}\to \sum \limits_{j}{w}_{j}{W}_{k}\left(\omega +{\omega }_{j},{R}_{2,j}^{{\prime}},TE\right),$$

assuming equal relaxation properties (T1 & T2) for each peak. If peaks have different relaxation properties, a separate phase graph calculation must be performed for each peak before summation.

#### Variable echo time and configuration state imaging

Our derivation does not impose restrictions on the type of readout employed in each TR. Thus, sequences with variable echo times and multi-echo sequences can equally be discussed^2,41^. This also includes sequences refocusing multiple configuration states, e.g. double- and triple-echo steady-state (DESS & TESS)^32,33^ or MRF employing configuration state imaging.

#### Arbitrary spoiling and k-space readout

We restricted spoiling to the through-slice direction, which allowed us to discuss slice profiles as a source for off-resonance artifacts in spoiled SSFP-based MRF. Using the same approach, however, we can also discuss arbitrary spoiling directions. In this case, three factors need to be considered separately: (1) the spatial distribution of the configuration states ${F}_{k}^{+}({\varvec{r}})$, which is a consequence of RF pulses, B1 + inhomogeneity, and variation of relaxation properties; (2) the coil sensitivity-weighted equilibrium magnetization distribution $C({\varvec{r}}){M}_{0}({\varvec{r}})$; and (3) Fourier encoding of the spatial frequency ${\varvec{k}}\boldsymbol{^{\prime}}$ with the associated time point of encoding $\tau ({{\varvec{k}}}^{\boldsymbol{{\prime}}}),$ which depends on the chosen acquisition trajectory. The signal Eq. (14) can then be generalized to

35 $${s}_{{\varvec{k}}^{\boldsymbol{\prime}},{\varvec{q}}}^{\left(n\right)} ={\int }_{\Omega }{\text{d}}^{3}r C\left({\varvec{r}}\right){M}_{0}\left({\varvec{r}}\right) \sum \limits_{k}{W}_{k}\left(\omega , {R}_{2}^{{\prime}},TE+\tau \left({{\varvec{k}}}^{\boldsymbol{{\prime}}}\right)\right){e}^{i(k-q){{\varvec{k}}}_{sp}\cdot {\varvec{r}}+{\text{i}}{\varvec{k}}^{\boldsymbol{\prime}}\cdot {\varvec{r}}}{F}_{k}^{+\left(n\right)}\left({\varvec{r}}\right).$$

Employing the convolution theorem, the signal is given by

36 $${s}_{{\varvec{k}}^{\boldsymbol{\prime}},q}^{(n)} =\sum \limits_{k}{W}_{k}\left(\omega , {R}_{2}^{{\prime}},TE+\tau \left({{\varvec{k}}}^{\boldsymbol{{\prime}}}\right)\right)\left(\mathcal{F}\left\{CM\right\}*\mathcal{F}\left\{{F}_{k-q}^{+\left(n\right)}\right\}\right)\left(k{{\varvec{k}}}_{sp}+{\varvec{k}}^{\boldsymbol{\prime}}\right).$$

Thus, interference of configurations can be equally formulated as a question of how well separated states are in k-space^42^, an insight already formulated and discussed in detail by Sobol and Gauntt^26^. In addition, effects of non-instantaneous acquisition, such as blurring through off-resonance and k-space filtering as a result of signal decay are equally captured through the dependency of the model on the sampling time-point $\tau ({{\varvec{k}}}^{\boldsymbol{{\prime}}})$.

#### Variable TR sequences

Central to the derivation is a constant phase increment $\phi =\omega \cdot TR+{k}_{sp}\cdot z$. This directly enforces a constant repetition-time TR, off-resonance $\omega$, and spoiling moment ${k}_{sp}$. Only in this case, the phase graphs for off-resonance and spoiling are equal. In addition, this leads to a constant phase advance angle $\phi$ per $TR$ and the phase graph will grow linearly with each iteration. Dropping this requirement, exponential growth ($\sim {3}^{n})$ of pathways quickly renders the SR-EPG intractable^8^. Dropping the constant $TR$ requirement, a phase graph needs to be simulated for each off-resonance frequency separately.

#### Equilibrium magnetization and coil sensitivity

Contrary to RF pulses, equilibrium magnetization and coil sensitivities enter as factors into the generalized signal equation (Eq. 35). From a state-mixing perspective, they can be represented as convolutions in k-space (Eq. 36). Hence, they generally lead to broadening of the configuration’s k-space representation and lead to increased mixing. Considering coil sensitivities as the only spatially varying component, they are expected to show minor state interference since they typically vary slowly in space. Contrary, equilibrium magnetization can vary strongly, e.g. at tissue interfaces or due to contrast agent uptake, leading to increased interference of configurations and thus off-resonance artifacts.

#### Continuous broadening of configuration states

Considering a single RF pulse within the small tip-angle approximation ($\normalsize \alpha \ll 1 \text{rad}$), Pauly’s k-space interpretation of slice-selective excitation can be employed to estimate the spatial frequencies excited^43^. The maximal frequency component in the excitation slice profile is then given by half the gradient moment of the slice selection gradient $\gamma G{T}_{ex}/2$. Thus, the minimal spoiling moment to fully dephase the transverse magnetization after a single RF pulse is given by

37 $${k}_{sp}\ge \gamma G{T}_{ex}/2=\frac{\pi \kappa }{\delta }.$$

Since RF pulses can be represented by independent matrix products in each spatial position, they also transform to convolutions in k-space. Hence, consecutive application of multiple RF pulses will generally lead to a broadening of the configuration state’s k-space populating at most the spatial frequency $n\pi \kappa /\delta$ after the ${n}$th RF pulse. This hinders complete spoiling unless signal cancellation due to interference is achieved (Figs. 7 and 8).

#### B1 + inhomogeneity

Transmit field inhomogeneity ($\Delta {B}_{1}^{+}$) affects the RF pulse matrices and thus is equally expected to lead to continuous configuration state broadening despite being slowly varying in space.

#### Strong spoiling and partitioned EPG

Ultimately, the continuous broadening of configuration states leads to the insight that spoiling as strong as possible is required to consistently dephase states below the noise floor^26^. Then, the partitioned EPG approximation is justified, which was recovered by the limit ${k}_{sp}\to \infty$ and is characterized by truly refocusing a single configuration.

#### Relaxation during RF

In the derivation of the effective RF pulse operator $RF(z)$, we have assumed negligible relaxation during excitation, which leads to a rotation matrix description with effective rotation angles. While this assumption holds true for most imaging sequences and tissues, short relaxation times and long RF pulses can lead to departure from this simplifying assumption. It is important to note, however, that the notion that RF pulses can be expressed as an effective matrix operator $RF(z)$ remains generally true since the solution of the Bloch equations can still be represented by a linear operator. Equally, the assertions made about the Hybrid Bloch-EPG formalism do not require a specific form of $RF(z)$. However, the RF pulse operator becomes parametrically dependent on ${T}_{1}$, ${T}_{2}$, and ${R}_{2}^{{\prime}}$, which can pose a significant computational burden for dictionary generation as RF pulses need to be calculated either on the fly or be pre-calculated for all combinations of relaxation parameters. Moreover, the RF operator is no longer a pure rotation matrix (${\text{det}}RF\left(z\right)\ne 1)$ implying that the description by effective flip-angle profiles is no longer possible. If and how this effective description of slice-selective RF pulses in the presence of relaxation can be extended, however, as well as a detailed analysis of dephasing effects during excitation, remains subject of future work.

### Implications for MR fingerprinting and configuration state imaging

A detailed discussion of the effect of slice profile and spoiling in the context of off-resonance artifacts has already been presented by Ostenson et al.^18^ We will therefore only discuss the specific findings in the context of configuration interference and configuration state imaging.

There are two regions in the test example MRF sequence, where magnitude and phase variation due to off-resonance are strongest: (1) the time at which longitudinal magnetization passes through zero during the recovery process (for F(0) and T1 = 800 ms, T2 = 50 ms (red curve), time index 25…40 in Fig. 6) and (2) high flip angle regions (time index 50…100). Following the argument of Sobol and our findings regarding interference of configuration states, an increase in off-resonance variation is related to increased contributions from unwanted states ${F}_{k\ne q}$ relative to the refocused state ${F}_{q}$.

In the case of configuration state imaging, a time-reversed spoiled SSFP-based fingerprinting sequence reading out the ${F}_{-1}$ configuration shows similar behavior to the ${F}_{0}$ imaging, albeit with generally reduced signal magnitude. For higher configurations (${F}_{+1}$ and ${F}_{-2}$), configuration state interference leads to pronounced signal magnitude variations (see Figs. 6 and 9). We attribute this to the generally low intensity of the ${F}_{+1}$ and ${F}_{-2}$ states compared to their neighboring ${F}_{0}$ and ${F}_{-1}$ states. Of note, while favorable spoiling moments exist, the valleys of low magnitude variations become steeper in Fig. 9, rendering them more susceptible to changes in the spoiling moment, e.g. due to local field inhomogeneities.

To the knowledge of the authors, MRF combined with configuration state imaging has so far not been published apart from time-reversed gradient-spoiled MRF (PSIF)^44^. While the combination of the two seems straightforward, the increase in off-resonance variation for higher order states as well as the difficulty of finding a common spoiling moment that works equally well across configurations might be one obstacle hindering successful implementation. Especially time-reversed spoiled-SSFP might be interesting in the realm of diffusion-sensitive MR Fingerprinting^34,35^.

**Figure 11:**
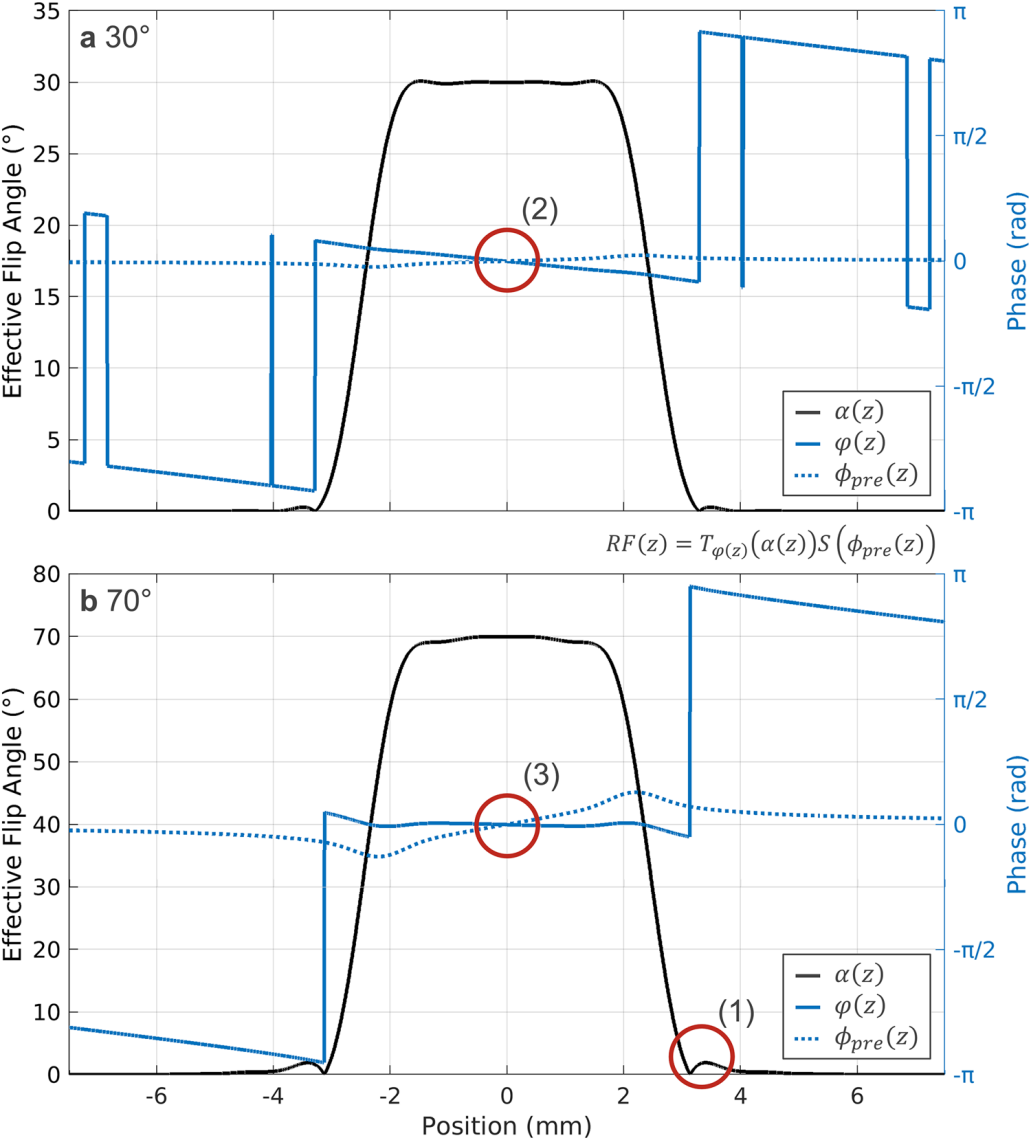
Effective RF pulse decomposition. Two exemplary slice profiles for (**a**) 30° and (**b**) 70° RF pulse decomposed into effective flip angle $\alpha \left(z\right)$ (black) and rf pulse phase profiles $\varphi (z)$ (blue, solid). The broken line corresponds to the effective pre-phaser ${\phi }_{pre}(z)$, which needs to be considered for storage and echo components of the pulse. (1) At high flip angle, slide lobes are amplified due to violation of the small tip angle approximation. (2) At low flip angles, the effective pulse phase is not flat as the gradient waveforms for the sinc pulses were optimized for the maximum flip angle (3) as is performed on contemporary scanner systems. For large flip angles, pronounced non-linearity in both phase terms arises at the boundary of the slice profiles.

To the knowledge of the authors, current MRF implementations employ RF pulse amplitude scaling to implement variable flip angles. This typically leads to an amplification of side lobes at high flip angles (Fig. 11(1)) and non-linear phase accrual over the slice profile (Fig. 11 (2) and (3)), which in turn result in increased off-resonance variation in high flip angle regions. Optimal design of RF pulses, pre-winder, and refocusing gradients might allow control over configuration interference and thus off-resonance artifacts in gradient-spoiled MRF.

The effective pulse representation (Eq. 5) allows to fully describe slice selective RF pulses through a position-dependent phase accrual ${\phi }_{pre}(z)$ and a hard pulse. Hence, together with the hybrid Bloch-EPG, we can formulate a model for slice profile corrections in DESS & TESS^45^ sequences using the analytical solutions for the transverse configuration states ${F}_{k}^{+}$ in steady state by Hänicke (use Eq. (21) in Hänicke, where ${S}_{n}$ denotes the steady state solutions of the signal magnitude of the configuration orders $n$)^46^. By defining

38 $${F}_{k}^{+}\left({z}_{i}\right)=\left\{\begin{array}{l}{\left.{S}_{n}\right|}_{\alpha \to \alpha ({z}_{i}),n=k} :k\ge 0\\ -{\left.{S}_{n}\right|}_{\alpha \to \alpha ({z}_{i}),n=k} :k<0\end{array}\right.,$$

we can evaluate the signal of the $q$th refocused echo in the presence of an arbitrary slice profile by

39 $${s}_{q}^{\left(n\right)}=C{M}_{0} \sum \limits_{k}{W}_{k}\left(\omega , {R}_{2}^{{\prime}},TE\right)\sum \limits_{i}{\mathcal{F}}_{k-q,i}\left({k}_{sp}\right){F}_{k}^{+}\left({z}_{i}\right){e}^{{\text{i}}{\phi }_{pre}\left({z}_{i}\right)k+{\text{i}}\varphi (z)}.$$

Finally, the hybrid Bloch-EPG allows to recover four dimensions analytically: $TE$, $\omega$, ${R}_{2}^{{\prime}}$, and the spoiling moment ${k}_{sp}$. Optimal parameter combinations of ${R}_{2}^{{\prime}}$ and $\omega$, could be determined by conventional fitting routines rather than being resolved through discrete atoms in a dictionary. This could help to reduce the dimensionality of the dictionaries and improve model-data consistency. Analytical access to ${k}_{sp}$ further allows for fast evaluation of optimal spoiling moments to improve sequence design and mitigate slice profile effects.

## Conclusion

The hybrid Bloch-EPG formalism proposed in this work utilizes a numerically calculated spatially-resolved EPG to analytically recover the MR signal dependency on echo time, microscopic dephasing, off-resonance, and spoiling moment. It is applicable to sequences with varying RF pulses but constant TR and spoiling moment, as they are commonly used in MR Fingerprinting. The hybrid Bloch-EPG formalism allows to seamlessly tune between spoiled and balanced SSFP-based MRF sequences. It was shown that off-resonance artifacts arise from interference of configuration states, which is a direct effect of incomplete spoiling due to non-rectangular slice profiles. The formalism retains a clear separation of configuration states and slice profiles, which allows for a more fundamental understanding of echo formation and artifact generation than alternative Bloch or slice-selective EPG calculations.

## Methods

The hybrid Bloch-EPG formalism was implemented in MATLAB 2020b (Mathworks, Natik, MA, USA) and compared to implementations of both Bloch simulation and slice-selective EPG. Hann-apodized sinc pulses with flip angles ${\alpha }_{n}$ according to Fig. 2a were generated with $\kappa =10$ and nominal slice thickness of 5 mm by amplitude scaling. Refocussing gradient area ${k}_{post}$ was calculated once for the maximal flip angle of 70° and the prewinder was defined as ${k}_{pre}=-\left(\gamma G{T}_{ex}+{k}_{post}\right)$. For each $n=1\dots 100$, effective RF pulse matrices $R{F}_{n}(z)$ were obtained using the hard pulse approximation. An adiabatic inversion pulse preceded the test sequence, which was simulated as an ideal pulse inverting the longitudinal magnetization only. Repetition time was assumed to be constant with $TR=15 \; {\text{ms}}$.

Bloch simulation was performed using the rotation operator algorithm and propagation of the magnetization through the hard pulse approximation (Eq. 4) in each repetition. The slice-selective EPG was implemented according to Ostenson et al. by performing the hard pulse approximation in the Fourier domain^18^. For comparison of the techniques in Fig. 3, rounding of spoiling, refocusing and prephasing moments to the state increment of the slice-selective EPG ($\Delta k=\gamma G\Delta t$) was performed as well as choosing 5119 isochromats and 145 temporal steps to ensure equivalence of the methods. For visualization purposes only, RF pulse phase alternation was not simulated.

For all other results and if not otherwise stated, plots depict phase graphs and signals after 91 RF pulses, i.e. time-point 90 within the fingerprinting train (Fig. 2a) with simulation parameters of $T1=800 \; {\text{ms}}$, $T2=50 \; {\text{ms}}$, ${R}_{2}^{{\prime}}=0$, $TE=0$, and $\omega =0$. The spatially-resolved EPG was simulated with 3001 isochromats, and a field-of-view of three times the nominal slice thickness.

For the comparison of hybrid Bloch-EPG magnetization profiles and the Bloch simulation (see Fig. 3), the normalized root-mean-square error (nRMSE) was calculated as follows

40 $$nRMS{E}^{\text{Bloch}}=\frac{\sqrt{{ \left \langle {\left|{M}_{+}^{\text{Bloch}}\left(z\right)-{M}_{+}^{\text{Hybrid} \; {\text{Bloch}}-{\text{EPG}}}\left(z\right)\right|}^{2} \right \rangle }_{z}\boldsymbol{ }}}{\left|{\langle {M}_{+}^{\text{Bloch}}\left(z\right)\rangle }_{z}\right|}$$

and the nRMSE for the ssEPG according to

41 $$nRMS{E}^{\text{ssEPG}}=\frac{\sqrt{{\left \langle {\left|{M}_{+}^{\text{ssEPG}}\left(k\right)-{M}_{+}^{\text{Hybrid} \; {\text{Bloch}}-\text{EPG}}\left(k\right)\right|}^{2} \right \rangle }_{k}\boldsymbol{ }}}{\left|{\langle {M}_{+}^{\text{Bloch}}\left(z\right)\rangle }_{z}\right|}.$$

Code to reproduce the figures in this paper can be found in the supporting material.

## Supplementary Information

Supplementary Information.

## Data Availability

All data presented was generated through custom code, which is shared as part of this publication.

## Code availability

Code to reproduce the findings of this paper can be found here: 10.5905/ethz-1007-437.
