# Supplementary material for: A unifying view on extended phase graphs and Bloch simulations for quantitative MRI
Source: Sci Rep. 2021 Oct 28;11:21289. doi: 10.1038/s41598-021-00233-6 (PMC8553818; doi:10.1038/s41598-021-00233-6)
Supplement: Supplementary file 1 — Supplementary Information. [file 41598_2021_233_MOESM1_ESM.docx]

A Unifying View on Extended Phase Graphs and Bloch Simulations for Quantitative MRI

Christian Guenthner^*1,2^, Thomas Amthor^2^, Mariya Doneva^2^, and Sebastian Kozerke^1^

^1^*Institute for Biomedical Engineering, University and ETH Zurich, Zurich, Switzerland
^2^Philips Research, Hamburg, Germany
^*^Corresponding Author*

# Appendix

## Appendix A: Matrix Operators for the Piecewise Solution of the Homogenized Bloch Equations

The matrix operators were taken from Weigel’s review on extended phase graphs and adapted to be conformant to the homogenized version of the Bloch equations.^8^

**Hard pulse** of flip angle $\alpha$ and phase $\varphi$

| $T_{\varphi}\left( \alpha\right)=\left( \begin{matrix} {\cos\left( \frac{\alpha}{2} \right)}^{2} & ⅇ^{2ⅈ\varphi}{\sin\left( \frac{\alpha}{2} \right)}^{2} & -ⅈⅇ^{ⅈ\varphi}\sin\left( \alpha\right) & 0 \\ ⅇ^{-2ⅈ\varphi}{\sin\left( \frac{\alpha}{2} \right)}^{2} & {\cos\left( \frac{\alpha}{2} \right)}^{2} & ⅈⅇ^{-ⅈ\varphi}\sin\left( \alpha\right) & 0 \\ -\frac{1}{2}ⅈⅇ^{-ⅈ\varphi}\sin\left( \alpha\right) & \frac{1}{2}ⅈⅇ^{ⅈ\varphi}\sin\left( \alpha\right) & \cos\left( \alpha\right) & 0 \\ 0 & 0 & 0 & 1 \end{matrix} \right)$ | (A.1) |
| --- | --- |

**Relaxation** **and** **Recovery** with relaxation time constant $T1$ and $T2$ and time $t$

| $R\left( t \right)=\left( \begin{matrix} \exp\left( -\frac{t}{T_{2}} \right) & 0 & 0 & 0 \\ 0 & \exp\left( -\frac{t}{T_{2}} \right) & 0 & 0 \\ 0 & 0 & \exp\left( -\frac{t}{T_{1}} \right) & 1-\exp\left( -\frac{t}{T_{1}} \right) \\ 0 & 0 & 0 & 1 \end{matrix} \right)$ | (A.2) |
| --- | --- |

**Phase Accrual** of phase $\phi$

| $S\left( \phi\right)=\left( \begin{matrix} \exp\left( i\phi\right) & 0 & 0 & 0 \\ 0 & \exp\left( -i\phi\right) & 0 & 0 \\ 0 & 0 & 1 & 0 \\ 0 & 0 & 0 & 1 \end{matrix} \right),$ | (A.3) |
| --- | --- |

with $\phi=\omega\cdot t+\boldsymbol{k}(t)\cdot\boldsymbol{r}$ and $\boldsymbol{k}(t)=\gamma\int_{0}^{t} \boldsymbol{G}\left( \tau\right)d\tau$, with $\gamma$ the gyromagnetic ratio.

## Appendix B: Derivation of the Solution to $\boldsymbol{y}^{\left( n \right)}=A^{\left( n \right)}S\boldsymbol{y}^{\left( n-1 \right)}$

Let $A^{(n)}\in\mathbb{C}^{4\times4}$ be an arbitrary set of complex matrix operators ($n=1\ldots N$) and $S$ be the following diagonal matrix

| $S:=\mathrm{diag}\left( e^{i\phi},e^{-i\phi},1,1 \right)$, | (B.1) |
| --- | --- |

where $\phi\mathbb{\in R}$ is constant but arbitrary. We are seeking the parametric solution $\boldsymbol{y}^{\left( N \right)}\left( \phi\right)$ of the recurrent matrix series

| $\boldsymbol{y}^{\left( n \right)}=A^{\left( n \right)}S\boldsymbol{y}^{\left( n-1 \right)},$ with $n=1\ldots N,$ | (B.2) |
| --- | --- |

and the initial condition $\boldsymbol{y}^{(0)}=\left( 0,0,1,1 \right)^{T}$.

The diagonal operator $S$ of Equation (B.1) can also be written as

| $S=\sum_{k=-\infty}^{\infty} e^{ik\phi}\mathrm{diag}\left( \delta_{k,1},\delta_{k,-1},\delta_{k,0}{,\delta}_{k,0} \right).$ | (B.3) |
| --- | --- |

In this expression, the parametric dependency is shifted into a scalar function, where the matrix operator itself is no longer an explicit function of $\phi$. This is convenient, since the matrix operator $A^{(n)}$ commutes with the exponential, allowing to write

| $\boldsymbol{y}^{\left( n \right)}=\sum_{k} e^{ik\phi}A^{\left( n \right)}\mathrm{diag}\left( \delta_{k,1},\delta_{k,-1},\delta_{k,0}{,\delta}_{k,0} \right)\boldsymbol{y}^{\left( n-1 \right)}.$ | (B.4) |
| --- | --- |

Here, summation over $k=-\infty\ldots\infty$ is implied. The form of the equation calls for the solution ansatz

| $\boldsymbol{y}^{(n)}=\sum_{k} e^{ik\phi}\boldsymbol{x}_{k}^{\left( n \right)},$ | (B.5) |
| --- | --- |

with $\boldsymbol{x}_{k}^{(0)}={\boldsymbol{y}^{\boldsymbol{(}0)}\delta}_{k,0}$. Insertion of this expression into Equation (B.2) yields

| $\sum_{k} e^{ik\phi}\boldsymbol{x}_{k}^{\left( n \right)}=\sum_{k^{'}} e^{ik^{'}\phi}A^{\left( n \right)}\mathrm{diag}\left( \delta_{k^{'},1},\delta_{k^{'},-1},\delta_{k^{'},0}{,\delta}_{k^{'},0} \right)\left( \sum_{k^{''}} e^{ik^{''}\phi}\boldsymbol{x}_{k^{''}}^{\left( n-1 \right)} \right).$ | (B.6) |
| --- | --- |

| $\Leftrightarrow\sum_{k} e^{ik\phi}\boldsymbol{x}_{k}^{(n)}=\sum_{k^{''},k^{'}} e^{i\left( k^{'}+k^{''} \right)\phi}A^{\left( n \right)}\mathrm{diag}\left( \delta_{k^{'},1},\delta_{k^{'},-1},\delta_{k^{'},0}{,\delta}_{k^{'},0} \right)\boldsymbol{x}_{k^{''}}^{(n-1)}.$ | (B.7) |
| --- | --- |

By equating the exponentials on either side, we find $k=k^{'}+k^{''}$, leading to

| $\boldsymbol{x}_{k}^{(n)}=\sum_{k^{'}} A^{\left( n \right)}\mathrm{diag}\left( \delta_{k^{'},1},\delta_{k^{'},-1},\delta_{k^{'},0}{,\delta}_{k^{'},0} \right)\boldsymbol{x}_{{k-k}^{'}}^{(n-1)},$ | (B.8) |
| --- | --- |

which can be equivalently written by shifting coefficients ($k^{'}\to k-k^{'}$) and using $\delta_{a,b}=\delta_{a-b,0}$ as

| $\boldsymbol{x}_{k}^{(n)}=\sum_{k^{'}} A^{\left( n \right)}\mathrm{diag}\left( \delta_{k,k^{'}+1},\delta_{k,k^{'}-1},\delta_{k,k^{'}}{,\delta}_{k,k^{'}} \right)\boldsymbol{x}_{k^{'}}^{(n-1)}.$ | (B.9) |
| --- | --- |

Since $A^{(n)}$ is independent of $k^{'}$, it can be exchanged with the summation yielding

| $\boldsymbol{x}_{k}^{\left( n \right)}=A^{\left( n \right)}\sum_{k^{'}} \mathrm{diag}\left( \delta_{k,k^{'}+1},\delta_{k,k^{'}-1},\delta_{k,k^{'}}{,\delta}_{k,k^{'}} \right)\boldsymbol{x}_{k^{'}}^{\left( n-1 \right)}.$ | (B.10) |
| --- | --- |

## Appendix C: The effect of spoiling and off-resonance on the signal in gradient-spoiled SSFP

We performed an experiment on a Philips Achieva 3T (Best, The Netherlands) system in a homogenous, spherical phantom (T1≈400 ms, T2≈340 ms) using a steady state gradient echo sequence (TR=9 ms, TE=4.5 ms) refocusing four configuration states (F_-2_ to F_+1_) in subsequent TRs (see Supp. Fig. 1a). The measurements were performed using Cartesian sampling and a constant flip angle of 30°. The spoiling moment was stepped between 2π/δ and 4⋅2π/δ in steps of 0.1⋅2π/δ. In addition, a linear field gradient of 2.5Hz/px was added by adjusting the linear shims resulting in a linear frequency ramp over the extent of the phantom. Exemplary images are shown in Supp. Fig. 1b. A region of interest (ROI, red) was drawn and averaged in left-right direction to obtain a single column for each of the configuration states shown in Supp. Fig. 1c. Supp. Fig. 1d displays the simulation results from the hybrid Bloch-EPG. For the simulation, the RF pulse was extracted from the measurements and simulated using a Bloch simulation on 5001 equidistantly spaced sampling points to obtain the RF(z) operator. The SR-EPG was calculated for 200 iterations to ensure steady state. The hybrid Bloch-EPG was then used to determine the signal’s dependency on both off-resonance and spoiling moment analytically.

**
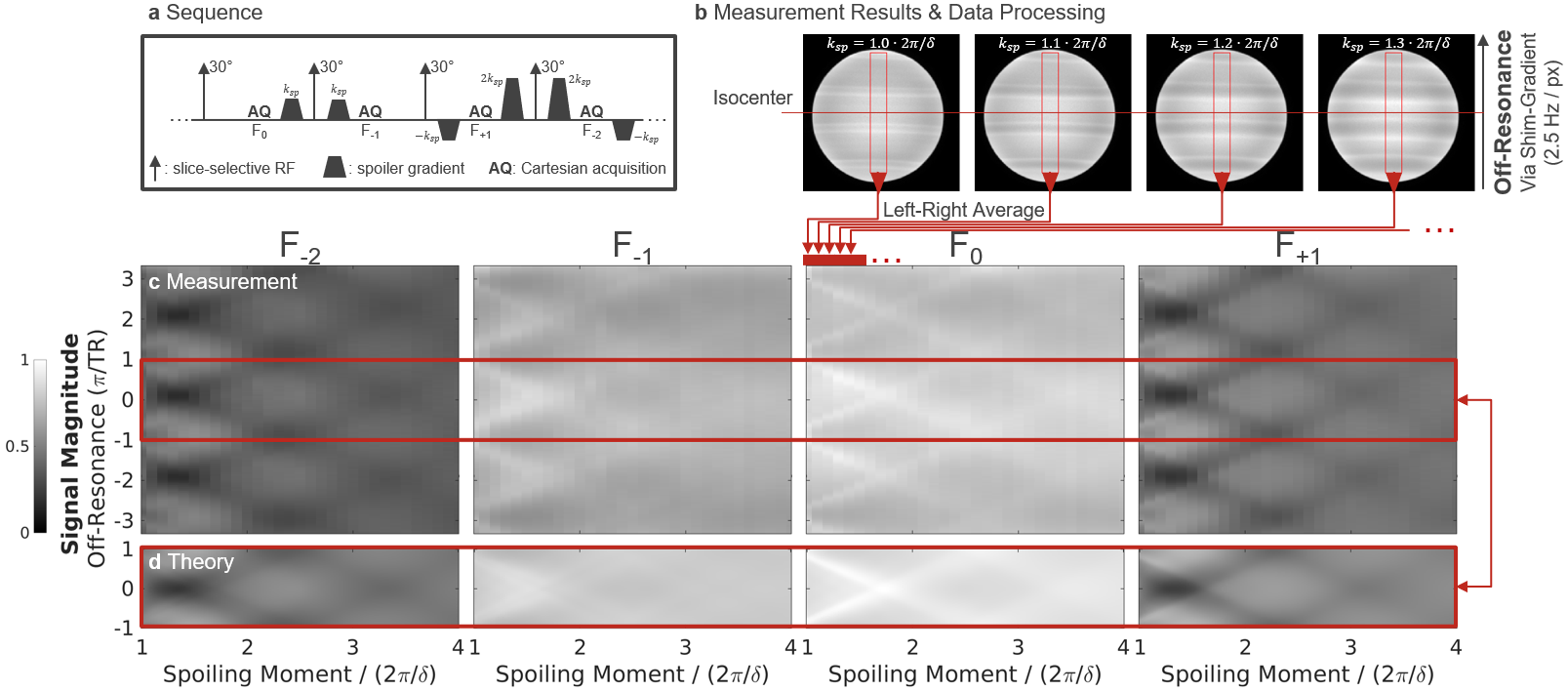
**

**Supplementary Figure 1:** Off-resonance and spoiling moment dependency of a spoiled steady-state free-precession sequence obtained through phantom measurements and compared to theoretical predictions of the hybrid Bloch-EPG formalism. **a** depicts the employed gradient-spoiled SSFP sequence with refocusing of four configuration states in subsequent TRs. The spoiling moment $\boldsymbol{k}_{\boldsymbol{sp}}$ was kept constant for each experiment but varied between measurements. **b** shows magnitude image resulting from four different spoiling moments $\boldsymbol{k}_{\boldsymbol{sp}}$. A linear off-resonance variation across the phantom was achieved through a linear shim gradient of 2.5Hz/pixel. The signal within the region of interest (red) was averaged left-to-right and constitutes one column in c. **c** shows the variation of signal magnitude as a function of both off-resonance (vertical axis) and spoiling moment (horizontal axis). **d** depicts the predictions of the proposed hybrid Bloch-EPG formalism.

The findings of Supp. Fig. 1c and d are in excellent qualitative agreement. There is a slight shift of the measurement in vertical (off-resonance) direction compared to theory, which most likely stems from a constant field offset as well as a slight shearing probably originating from field-drift between consecutive measurements.
